# Tumor-associated macrophage reprogramming in cancer: recent advances in resistance mechanisms and therapy

**DOI:** 10.3389/fimmu.2026.1920004

**Published:** 2026-10-28

**Authors:** Abdullah Farhan Saeed, Muhammad Naim Ashraf, Muhammad Zaheer Iqbal

**Affiliations:** 1 Department of Internal Medicine, University of Michigan, Ann Arbor, MI, United States; 2 Department of Pathology, University of Lahore, Lahore, Punjab, Pakistan; 3 Department of Biological Sciences, Superior University, Lahore, Punjab, Pakistan

**Keywords:** immune checkpoint blockade, macrophage plasticity, myeloid immunotherapy, TAM reprogramming, therapy resistance, tumor microenvironment, tumor-associated macrophages

## Abstract

Tumor-associated macrophages (TAMs) are abundant, heterogeneous, and spatially organized immune cells that regulate cancer progression, immune evasion, metastasis, and therapeutic resistance. Single-cell and spatial technologies have revealed diverse TAM states shaped by ontogeny, tumor type, disease stage, cytokines, hypoxia, metabolism, epigenetic remodeling, extracellular matrix architecture, stromal interactions, extracellular vesicles, microbiome-derived signals, and therapy-induced damage. Bidirectional cancer-TAM crosstalk evolves from tumor initiation through progression and recurrence, generating tumor-specific resistance programs that suppress lymphocytes, impair antigen presentation and phagocytosis, promote angiogenesis and metabolic restriction, remodel stroma, mediate tolerogenic efferocytosis, and sustain stem-cell, metastatic, dormancy, and regenerative niches. Therapeutic strategies include blocking macrophage recruitment or survival, activating CD40/TLR/STING signaling, targeting PI3Kγ and STAT pathways, restoring phagocytosis, modulating efferocytosis, and applying metabolic, epigenetic, stromal, vascular, nanoparticle, RNA-based, microbiome-directed, and engineered-macrophage approaches. Randomized trials have established the efficacy of CSF1R inhibition in selected CSF1-driven, macrophage-dependent tumors, whereas most other TAM- or myeloid-directed strategies in conventional solid tumors have demonstrated only preliminary clinical activity or pharmacodynamic modulation without consistent durable benefit. Off-target toxicity, spatial heterogeneity, dynamic plasticity, preclinical-clinical discordance, and combination-treatment optimization remain major barriers. Biomarker-guided patient selection, longitudinal monitoring, tumor-specific targeting, and rationally sequenced combinations will be essential for converting tumor-supportive TAMs into durable antitumor effectors.

## 1 Introduction

TAMs are among the most abundant and functionally diverse immune cells in the tumor microenvironment (TME) (1, 2). They regulate cancer progression, immune evasion, angiogenesis, metastasis, and therapeutic response. TAMs are recruited, expanded, and functionally educated by tumor cells, stromal cells, endothelial cells, cancer-associated fibroblasts (CAFs), dying cells, metabolites, extracellular matrix (ECM) components, and therapy-induced damage signals. Their biological behavior is shaped by tumor type, tissue origin, disease stage, anatomical niche, treatment exposure, and systemic host factors (3–5).

As per previous studies, macrophage polarization has been described using the M1/M2 framework, in which M1-like macrophages were viewed as inflammatory and tumor-restraining, whereas M2-like macrophages were considered immunosuppressive and tumor-promoting (3, 5–9). This model remains useful as a broad conceptual shorthand, but it does not portray the complexity of macrophage states in human tumors. Single-cell RNA sequencing (scRNA-seq), spatial transcriptomics, multiplex imaging, lineage tracing, and metabolomics have revealed TAM subsets specialized in antigen presentation, immune suppression, angiogenesis, lipid handling, matrix remodeling, hypoxia adaptation, efferocytosis, and tissue repair. Therefore, TAM abundance alone is often insufficient to predict prognosis or treatment response; macrophage phenotype, ontogeny, spatial localization, and treatment-induced dynamics are more informative (8–10).

TAMs arise from both embryonically derived tissue-resident macrophages and bone marrow-derived monocytes. Their recruitment and survival are regulated by pathways such as C-C motif chemokine ligand 2–C-C chemokine receptor 2 (CCL2–CCR2), colony-stimulating factor 1–colony-stimulating factor 1 receptor (CSF1–CSF1R), C-X-C motif chemokine ligand 12–C-X-C chemokine receptor 4 (CXCL12–CXCR4), C-C motif chemokine ligand 5–C-C chemokine receptor 5 (CCL5–CCR5), vascular endothelial growth factor–vascular endothelial growth factor receptor (VEGF–VEGFR), and angiopoietin 2–TIE2 receptor tyrosine kinase (ANGPT2–TIE2) (11–14). Once established in tumor niches, TAMs secrete interleukin-10 (IL-10), transforming growth factor beta (TGF-β), interleukin-6 (IL-6), VEGF, epidermal growth factor (EGF), hepatocyte growth factor (HGF), insulin-like growth factor 1 (IGF-1), matrix metalloproteinases (MMPs), cathepsins, and other mediators that suppress cytotoxic lymphocytes, support tumor-cell survival, stimulate angiogenesis, remodel ECM, and generate immune-excluded regions (15–20).

TAMs contribute to resistance against chemotherapy, radiotherapy, targeted therapy, anti-angiogenic therapy, and immune checkpoint blockade (ICB). After cytotoxic injury, macrophages clear apoptotic tumor cells through efferocytosis and initiate wound-healing programs that may suppress inflammation and support residual tumor-cell regrowth. In hypoxic, acidic, metabolically stressed, and stromal-rich regions, TAMs reinforce immune suppression, abnormal vascularization, ECM barriers, and regenerative niches that favor recurrence and metastasis (2, 9).

Importantly, TAM states and resistance mechanisms vary across cancer types and evolve from tumor initiation through progression, treatment, and recurrence. Bidirectional cancer-TAM crosstalk enables malignant cells, including therapy-resistant populations, to recruit and educate immunosuppressive macrophages, which subsequently reinforce tumor survival and progression through adaptive immune, metabolic, angiogenic, and stromal programs (21–24). This heterogeneity and plasticity limit the durability of single-target interventions and contribute to discrepancies between preclinical efficacy and clinical outcomes. Although completed and ongoing trials have evaluated CSF1R and CCR2 inhibitors, CD40 agonists, PI3Kγ inhibitors, CD47-SIRPα blockade, receptor-directed therapies, and engineered macrophages, clinical translation remains constrained by off-target toxicity, compensatory myeloid pathways, patient heterogeneity, and unresolved questions regarding combination dosing, timing, and sequencing (25–29).

Therapeutic strategies targeting TAMs have evolved from broad macrophage depletion toward more precise reprogramming (8, 9, 30, 31). Current approaches include CSF1R inhibition, blockade of monocyte recruitment, activation of CD40, Toll-like receptor (TLR), and stimulator of interferon genes (STING) pathways, phosphoinositide 3-kinase gamma (PI3Kγ) inhibition, restoration of phagocytosis through CD47–signal regulatory protein alpha (SIRPα) or related checkpoint blockade, metabolic and epigenetic modulation, nanoparticle-based delivery, extracellular vesicle (EV)-based strategies, and engineered macrophage therapies. These strategies are most likely to succeed when applied in biomarker-selected patients and combined with ICB, chemotherapy, radiotherapy, anti-angiogenic therapy, vaccines, or adoptive cell therapy (25, 32–36). This review describes the molecular, metabolic, spatial, tumor-specific, and stage-dependent mechanisms governing TAM plasticity and bidirectional cancer-TAM crosstalk; defines their roles in immune evasion and therapeutic resistance; evaluates preclinical and clinical evidence for TAM-targeted and engineered-macrophage therapies; and discusses biomarker-guided strategies for achieving durable antitumor macrophage reprogramming.

This narrative review was informed by targeted literature searches and reference-list screening. We prioritized recent studies addressing TAM ontogeny, functional heterogeneity, spatial organization, therapy resistance, therapeutic targeting, and clinical translation, while retaining seminal earlier studies where necessary to establish key concepts. Original mechanistic studies and primary clinical-trial reports were prioritized over secondary summaries where available. Because this was a narrative rather than a systematic review, no formal meta-analysis or risk-of-bias assessment was performed.

## 2 TAM origin, plasticity, and functional diversity

TAM function is determined by a combination of developmental origin, recruitment pathway, local education, metabolic state, spatial niche, and therapy exposure (9, 37, 38). Here we first outline how TAM populations are generated and diversified, then explain the major molecular and environmental programs that shape their behavior.

### 2.1 Recruitment, ontogeny, and TAM subset formation

A central determinant of TAM biology is the persistent recruitment of circulating monocytes and their differentiation within tumor niches. Tumor cells, CAFs, endothelial cells, stromal immune cells, and damaged tissues produce chemokines that attract monocytes from blood and bone marrow (10, 38–40). The CCL2–CCR2 axis is one of the best-characterized pathways driving the recruitment of inflammatory monocytes into tumors. Once recruited, monocytes differentiate into macrophages under the influence of cytokines, growth factors, oxygen tension, nutrient availability, ECM architecture, and direct cell-cell interactions (7, 41).

The CSF1–CSF1R pathway is another major regulator of macrophage survival, differentiation, and protumoral programming. CSF1R signaling activates PI3K-Akt, extracellular signal-regulated kinase (ERK), and signal transducer and activator of transcription 3 (STAT3) pathways, supporting macrophage survival, proliferation, angiogenic factor production, matrix remodeling, and immunosuppressive polarization (1, 8, 9, 41).

High CSF1 or CSF1R expression is associated with increased TAM accumulation, poor prognosis, and therapy resistance in several solid tumors. CSF1R inhibition can deplete or reprogram macrophages in preclinical models, but clinical responses as monotherapy have generally been unclear because tumors can activate compensatory recruitment and survival pathways (26, 42–44).

Additional chemokine and growth-factor axes contribute to TAM heterogeneity. CXCL12-CXCR4 signaling promotes myeloid localization in perivascular and immune-excluded niches, particularly in stromal-rich tumors (45–48). CCL5-CCR5 signaling recruits monocytes and regulatory immune populations in breast, pancreatic, and colorectal cancers (49). ANGPT2-TIE2 signaling enriches proangiogenic TIE2-positive macrophages near blood vessels, supporting neovascularization, vascular leakage, tumor-cell intravasation, and metastatic dissemination. C-X3-C motif chemokine ligand 1–C-X3-C motif chemokine receptor 1 (CX3CL1-CX3CR1) may regulate macrophage retention and survival in selected tissue settings (21, 50–52).

TAM plasticity is also influenced by ontogeny. TAMs are derived from both embryonically seeded tissue-resident macrophages and bone marrow-derived monocytes. These lineages differ in epigenetic memory, self-renewal capacity, receptor expression, niche preference, and therapeutic vulnerability (53–55). In glioblastoma, for instance, yolk sac-derived microglia coexist with infiltrating monocyte-derived macrophages. Microglia and recruited macrophages occupy distinct spatial niches and may respond differently to radiotherapy, corticosteroids, targeted therapy, and immunotherapy. Transmembrane protein 119 (TMEM119) and purinergic receptor P2Y12 (P2RY12) are commonly associated with microglia, whereas CCR2 and integrin alpha 4/CD49d help identify infiltrating monocyte-derived macrophages, although marker expression can shift under inflammatory or malignant conditions (56, 57).

Resident macrophages also contribute to tumor biology in organ-specific ways. Kupffer cells in the liver, alveolar macrophages in the lung, peritoneal macrophages in ovarian cancer, pancreatic macrophages in pancreatic ductal adenocarcinoma (PDAC), and bone marrow macrophages in skeletal metastases may acquire specialized tumor-supportive or tumor-suppressive states (27, 41, 58–60). During tumor evolution, tissue-resident macrophages may dominate early lesions, whereas monocyte-derived TAMs often expand during progression, recurrence, metastasis, and therapy-induced inflammation (8, 22, 61).

Single-cell studies have identified multiple TAM subsets, including antigen-presenting macrophages, interferon-stimulated macrophages, lipid-associated macrophages, angiogenic macrophages, matrix-remodeling macrophages, proliferating macrophages, immunoregulatory macrophages, and disease-associated populations. These populations are marked by triggering receptor expressed on myeloid cells 2 (TREM2), macrophage receptor with collagenous structure (MARCO), secreted phosphoprotein 1 (SPP1), apolipoprotein E (APOE), complement C1q C chain (C1QC), ecto-5′-nucleotidase/CD73, CD163, or heme oxygenase 1 (HMOX1) (6, 40, 62). These TAM subsets are not necessarily fixed lineages. Many can interconvert under environmental pressure, including hypoxia, cytokine shifts, metabolic stress, TLR or STING activation, CSF1R blockade, histone deacetylase (HDAC) inhibition, radiotherapy, chemotherapy, and ICB. Thus, TAM classification reflects both developmental origin and local education. Ontogeny provides a framework, but the TME and therapy exposure actively remodel macrophage state over time (63–67). Together, TAM heterogeneity reflects the integration of macrophage ontogeny, monocyte recruitment pathways, local TME-derived education signals, and therapy-induced remodeling into dynamic functional states (**Figure 1**).

**Figure 1:**
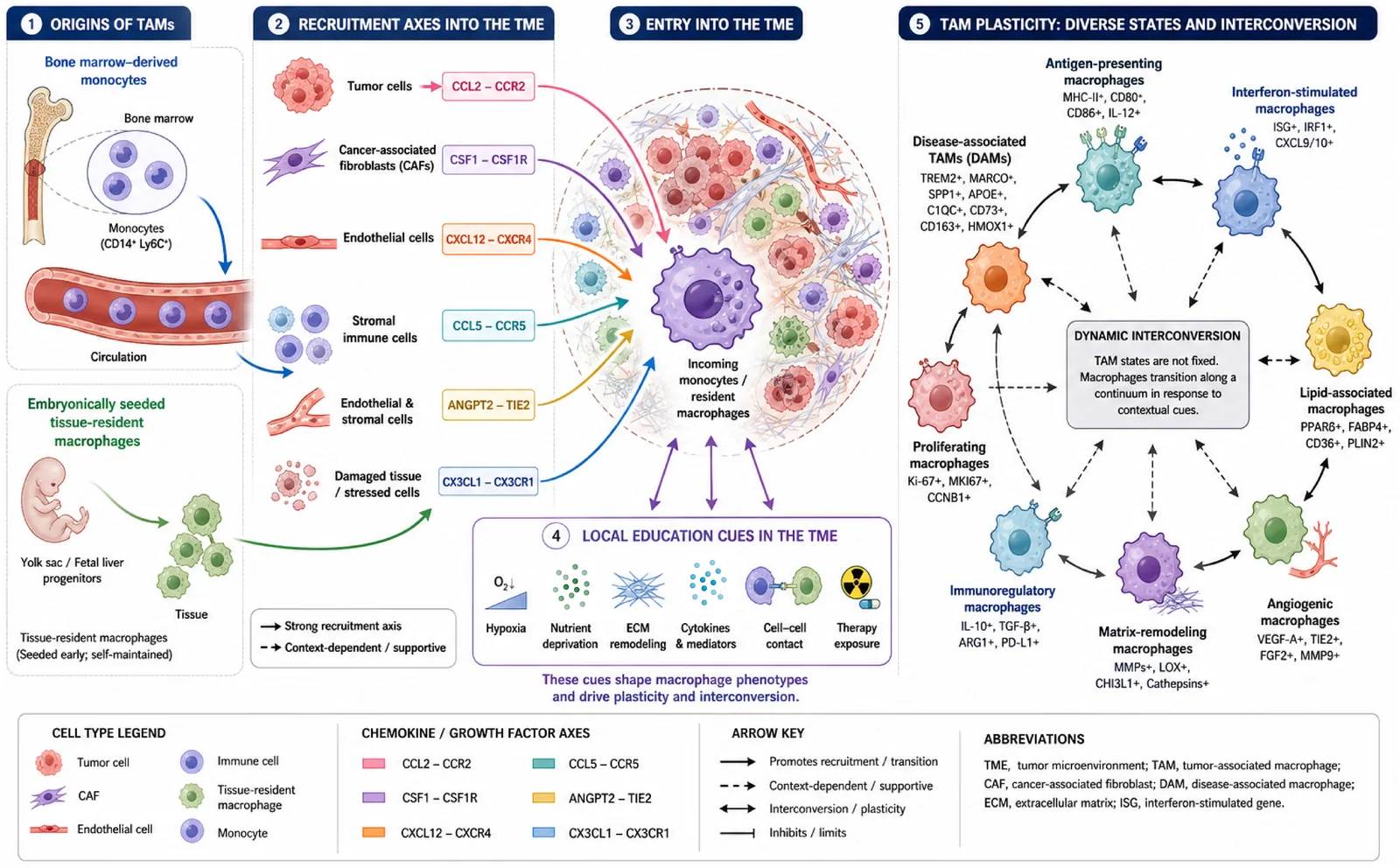
TAM origin, recruitment, and plasticity in the TME. This figure illustrates how TAMs arise from bone marrow-derived monocytes and embryonically seeded tissue-resident macrophages and subsequently diversify within the TME. Chemokine and growth-factor axes-including CCL2-CCR2, CSF1-CSF1R, CXCL12-CXCR4, CCL5-CCR5, ANGPT2-TIE2, and CX3CL1-CX3CR1-regulate macrophage recruitment, retention, and spatial positioning. Local cues, including hypoxia, cytokines, ECM architecture, nutrient availability, and therapy exposure, collectively shape TAM phenotypes. These inputs generate multiple interconverting functional states, including antigen-presenting, interferon-stimulated, lipid-associated, angiogenic, matrix-remodeling, immunoregulatory, proliferative, and disease-associated programs. Illustrative markers include TREM2, MARCO, SPP1, APOE, C1QC, CD73, CD163, and HMOX1. The figure emphasizes that TAM classification reflects both ontogeny and dynamic environmental programming.

### 2.2 Cytokine, growth-factor, and transcriptional control of TAM states

After recruitment or local expansion, TAMs are educated by a complex cytokine and growth-factor milieu produced by tumor cells, CAFs, endothelial cells, lymphocytes, dying cells, and innate immune populations (23, 41, 68, 69). These signals converge on Janus kinase-signal transducer and activator of transcription (JAK-STAT), PI3K-Akt-mechanistic target of rapamycin (mTOR), mitogen-activated protein kinase (MAPK), nuclear factor kappa B (NF-κB), SMAD, interferon-regulated, hypoxia-inducible factor (HIF)-dependent, and nuclear receptor pathways to generate context-specific macrophage states (9, 70–73).

Protumoral macrophage programs are commonly reinforced by IL-4, IL-13, IL-10, TGF-β, CSF1, IL-6, prostaglandin E2 (PGE2), VEGF, and tumor-derived growth factors. IL-4 and IL-13 activate STAT6 and promote tissue repair, scavenger receptor expression, matrix remodeling, and arginase-associated programs (1, 2, 27, 62, 74–76). IL-10 and IL-6 activate STAT3, suppress inflammatory cytokine production, reduce antigen presentation, enhance programmed death-ligand 1 (PD-L1) expression, and stabilize immunosuppressive TAM phenotypes. TGF-β signals through SMAD-dependent and SMAD-independent pathways to induce immune suppression, ECM remodeling, fibrosis, epithelial-mesenchymal transition (EMT), and exclusion of cytotoxic lymphocytes from tumor nests (8, 16, 77–80).

By contrast, interferon gamma (IFN-γ), granulocyte-macrophage colony-stimulating factor (GM-CSF), tumor necrosis factor (TNF), type I interferons (IFNs), and selected danger-associated signals can promote antigen presentation, nitric oxide production, IL-12 release, chemokine secretion, and cytotoxic T-cell recruitment. STAT1, interferon regulatory factor 1 (IRF1), IRF5, IRF8, and NF-κB are associated with inflammatory and antigen-presenting macrophage programs (7, 81–86). However, inflammatory states within tumors are often transient. Chronic antigen exposure, persistent cytokine stimulation, hypoxia, metabolic stress, and checkpoint signaling induce negative feedback regulators, including suppressor of cytokine signaling (SOCS) proteins, IL-10, PD-L1, indoleamine 2,3-dioxygenase (IDO), arginase-1 (ARG1), and other suppressive mediators (16, 87, 88).

At the transcriptional level, cytokine and growth-factor pathways are integrated by state-determining transcription factors, including STAT1, STAT3, STAT6, NF-κB, IRF1, IRF5, IRF8, HIF-1α, HIF-2α, activator protein 1 (AP-1), CCAAT/enhancer-binding protein beta (C/EBPβ), peroxisome proliferator-activated receptor gamma (PPARγ), liver X receptor (LXR), sterol regulatory element-binding protein (SREBP), nuclear factor erythroid 2-related factor 2 (Nrf2), and SMAD-family proteins. These transcription factors regulate antigen presentation, phagocytosis, angiogenesis, matrix remodeling, immune suppression, and metabolism (2, 89, 90). This cytokine-transcription factor network provides the molecular basis for many resistance mechanisms discussed later. In particular, IL-10, TGF-β, IL-6/STAT3, CSF1R, and HIF-dependent programs repeatedly emerge as central drivers of suppressive and therapy-refractory TAM states (7, 79, 91–94).

### 2.3 Metabolic and epigenetic reprogramming of TAMs

TAMs exhibit extensive metabolic plasticity, adjusting energy production, biosynthesis, redox balance, and immune function in response to hypoxia, nutrient deprivation, acidosis, oxidative stress, and competition with tumor cells. In tumors, macrophages may coexpress glycolytic, oxidative, lipid-handling, and stress-adaptation programs depending on niche localization and activation history (43, 95, 96).

Hypoxia is a major driver of TAM metabolism. HIF-1α and HIF-2α regulate macrophage responses to oxygen deprivation and induce the expression of genes involved in glycolysis, angiogenesis, tissue remodeling, and immunosuppression (97–99). Hypoxic TAMs often express VEGF, MMPs, ARG1, IL-10, and other factors that promote abnormal vasculature, tumor invasion, and T-cell dysfunction. Lactate produced by glycolytic tumor cells further shapes TAM plasticity by activating HIF-dependent and epigenetic programs that promote VEGF expression, arginine metabolism, angiogenesis, and immunosuppressive cytokine production (9, 100, 101).

Amino acid metabolism is also central to TAM-mediated immunosuppression. ARG1^hi^ TAMs deplete L-arginine, limiting T-cell proliferation and reducing nitric oxide production (41, 102). IDO and tryptophan 2,3-dioxygenase (TDO) catabolize tryptophan into kynurenine, which suppresses effector T cells, promotes regulatory T-cell (Treg) differentiation, activates aryl hydrocarbon receptor (AhR) signaling, and skews macrophages toward tolerogenic states. Glutamine metabolism supports macrophage survival, redox homeostasis, and polarization, and several studies suggest that targeting glutamine-dependent pathways may promote inflammatory macrophage reprogramming and enhance T-cell infiltration (103–105).

Lipid metabolism strongly influences TAM function. Lipid-laden TAMs can acquire scavenger receptor expression, oxidative metabolism, ferroptosis-related stress programs, and immunosuppressive features. CD36-mediated lipid uptake, fatty acid oxidation (FAO), cholesterol handling, lipid droplet formation, and prostaglandin production all tune macrophage phenotype (62, 106, 107). In several tumor models, lipid-loaded or FAO-dependent macrophages exhibit protumoral traits, whereas inhibition of lipid metabolic pathways can restrict macrophage-mediated suppression and sensitize tumors to therapy. Iron metabolism and ferroptosis-related pathways add another layer, as macrophages can store, recycle, or release iron, influencing tumor-cell proliferation, oxidative stress, and sensitivity to ferroptosis-inducing therapies (41, 108–110).

Metabolic reprogramming is closely linked to epigenetic regulation. TAM plasticity can be stabilized or reversed by chromatin accessibility, histone modifications, DNA methylation, and transcriptional memory (111–113). HDACs, DNA methyltransferases (DNMTs), bromodomain and extraterminal domain (BET) proteins, and chromatin-remodeling complexes regulate accessibility at loci involved in IL-10 production, arginine metabolism, antigen presentation, VEGF expression, and matrix remodeling. Class IIa HDACs, including HDAC4, have emerged as regulators of protumoral macrophage states, and HDAC inhibition can promote inflammatory, phagocytic TAM phenotypes in various preclinical models (114–117).

Tumor-derived metabolites can directly regulate macrophage epigenetics. Lactate, 2-hydroxyglutarate, succinate, fumarate, acetyl-coenzyme A (acetyl-CoA), and nicotinamide adenine dinucleotide (NAD)-dependent pathways can alter histone acetylation, histone methylation, and DNA methylation (118–120). Prior inflammatory exposure also influences macrophage responses through trained immunity or tolerance. Acute stimulation can prime enhanced inflammatory responses, whereas chronic stimulation can produce hyporesponsive, suppressive, or repair-oriented states. This has important implications for therapies involving TLR agonists, STING agonists, radiotherapy, oncolytic viruses, or cytokine modulators (121–124).

Post-transcriptional regulation through non-coding RNAs and EVs further contributes to TAM plasticity (125, 126). Tumor-derived EVs carry microRNAs (miRNAs), long non-coding RNAs (lncRNAs), circular RNAs (circRNAs), proteins, metabolites, and lipids that reprogram macrophages toward immunosuppressive, angiogenic, or metastatic phenotypes. TAM-derived EVs reciprocally affect tumor cells by transferring miRNAs, enzymes, cytokines, or proteins that promote EMT, stemness, migration, invasion, drug efflux, DNA repair, metabolic adaptation, and resistance to chemotherapy or targeted therapy (127–129).

### 2.4 Spatial, mechanical, organ-specific, and systemic regulation

TAM plasticity is profoundly spatial. Macrophages located in hypoxic tumor cores, perinecrotic regions, invasive margins, perivascular niches, lymphatic zones, stromal-rich areas, and immune-excluded compartments experience distinct oxygen levels, metabolites, cytokines, mechanical forces, and neighboring cell types (21, 38, 130, 131). As a result, functionally distinct TAM subsets can coexist within the same tumor, and their spatial location may be more predictive of therapy response than total macrophage abundance (28, 68).

Hypoxic and perinecrotic regions often enrich macrophages expressing HIF-regulated, angiogenic, glycolytic, and tissue-remodeling programs. These TAMs produce VEGF, MMPs, ARG1, IL-10, and other mediators that support abnormal vasculature, immune suppression, tissue invasion, and resistance to cytotoxic therapy (29, 41, 114, 132). Perivascular macrophages promote angiogenesis, vascular permeability, tumor-cell intravasation, and metastatic dissemination. At invasive margins, TAMs interact with tumor cells and CAFs to remodel the ECM, promote EMT, and create migratory tracks that facilitate invasion (1, 133).

ECM composition and stiffness also regulate macrophage phenotype. Dense collagen, hyaluronan, fibronectin, lysyl oxidase (LOX) activity, matrix crosslinking, and tissue stiffness activate integrin signaling, focal adhesion kinase (FAK), mechanotransduction, and yes-associated protein/transcriptional coactivator with PDZ-binding motif (YAP/TAZ)-related pathways in stromal cells and macrophages. These signals reinforce tissue-repair-like TAM states and physically restrict T-cell entry (93, 134–136). CAFs amplify these effects by producing TGF-β, CXCL12, IL-6, periostin, LOX, osteopontin, and dense collagenous ECM (137–140).

Tumor-specific tissue environments generate distinct TAM compositions and resistance mechanisms. In lung cancer, resident alveolar macrophages and recruited monocyte-derived macrophages can coexist with interferon-responsive, antigen-presenting, inflammatory, lipid-associated, angiogenic, and immunosuppressive states. Compared with strongly desmoplastic tumors such as PDAC, some lung cancers retain relatively pro-inflammatory or antigen-presenting macrophage populations that may support responses to ICB. However, lung-cancer TAMs are not uniformly inflammatory; histological subtype, smoking exposure, oncogenic drivers, disease stage, hypoxia, and treatment history can promote PD-L1-positive, TREM2-positive, angiogenic, or lipid-associated suppressive states. These differences support biomarker-guided reprogramming or checkpoint-based combinations rather than indiscriminate macrophage depletion (24, 81, 141).

By contrast, PDAC is commonly dominated by hypoxic, fibrotic, tissue-repair, and immunosuppressive TAM programs maintained by CSF1, CCL2, IL-6, TGF-β, CXCL12, lactate, CAFs, and dense ECM. These macrophages contribute to T-cell exclusion, impaired drug penetration, efferocytosis, stemness, and resistance to chemotherapy and ICB. TAM-directed treatment in PDAC may therefore require simultaneous stromal or vascular normalization, inhibition of monocyte recruitment or suppressive signaling, and activation of antigen presentation and T-cell immunity (142–145). Glioblastoma presents a different challenge because resident microglia and infiltrating monocyte-derived macrophages occupy distinct niches and may respond differently to radiotherapy and myeloid-directed therapies (39, 146, 147). In ovarian cancer, peritoneal and ascites-associated macrophages support spheroid survival, lipid transfer, metastatic implantation, and immune suppression, making locoregional macrophage targeting a potentially relevant strategy (102, 148, 149). Thus, TAM-directed treatment should be matched to cancer type, macrophage ontogeny, dominant functional state, spatial niche, and resistance mechanism rather than applied uniformly across tumors.

Systemic host factors also influence TAM states. The gut and intratumoral microbiome regulate myeloid cell development, cytokine tone, interferon signaling, pattern recognition receptor (PRR) activation, and responsiveness to ICB (150–152). Antibiotic exposure, dysbiosis, diet, microbial metabolites, obesity, aging, metabolic disease, systemic inflammation, circadian rhythms, glucocorticoids, adrenergic signaling, and sex hormones can alter monocyte production, macrophage lipid metabolism, cytokine responses, phagocytosis, antigen presentation, and resolution programs. These systemic influences help explain why patients with similar tumor genotypes can exhibit different TAM landscapes and treatment outcomes (127, 153, 154).

### 2.5 Temporal evolution, cancer-TAM crosstalk, and therapy-induced TAM plasticity

TAM composition and function evolve throughout tumor development. During tumor initiation, tissue-resident macrophages respond to oncogenic stress, tissue injury, dying cells, and inflammatory signals and may initially eliminate transformed cells or restrain excessive inflammation. Under persistent injury, however, repair-oriented macrophage programs can support the survival and expansion of premalignant cells. During early tumor growth, cancer-cell-derived CSF1, CCL2, IL-34, VEGF, cytokines, metabolites, and EVs recruit circulating monocytes and promote their differentiation into angiogenic, matrix-remodeling, and immunoregulatory TAMs. As tumors progress, chronic hypoxia, acidosis, nutrient competition, fibrosis, and sustained cytokine signaling enrich immunosuppressive, efferocytic, lipid-associated, and tissue-repair-associated states that inhibit antitumor immunity and facilitate invasion, metastasis, and therapeutic resistance (22, 28, 69, 151, 155). These temporal differences have therapeutic implications: early lesions may benefit from preserving tumor-restrictive resident macrophages, whereas advanced or recurrent tumors may require recruitment blockade, selective depletion, or reprogramming of established suppressive TAM populations.

Cancer cells and TAMs participate in bidirectional signaling that co-evolves during malignant progression and treatment. Cancer cells educate macrophages through CSF1, CCL2, IL-34, IL-6, TGF-β, VEGF, lactate, prostaglandins, and EV cargo, whereas TAMs reciprocally promote cancer-cell survival, EMT, stemness, invasion, and drug resistance through IL-6, TGF-β, EGF, HGF, IGF-1, WNT ligands, proteases, and EVs (70, 155–159). Chemotherapy-surviving or therapy-resistant cancer cells can alter their secretome and metabolic output to recruit monocytes and expand immunosuppressive TAM populations. These TAMs subsequently protect residual cancer cells by suppressing cytotoxic immunity, clearing apoptotic cells through tolerogenic efferocytosis, activating survival and repair pathways, and rebuilding angiogenic and stromal niches. Resistance therefore emerges through reciprocal adaptation between cancer cells and macrophages rather than through cancer-cell-intrinsic mechanisms alone.

Cancer therapies further intensify this temporal plasticity. Chemotherapy, radiotherapy, targeted therapy, anti-angiogenic therapy, PARP inhibitors, ICB, oncolytic viruses, CAR-T therapy, and emerging CAR-macrophage therapy can reshape macrophage states. In some contexts, treatment induces immunogenic cell death (ICD), type I interferon signaling, antigen presentation, phagocytosis, and macrophage-mediated tumor clearance. In others, therapy recruits monocytes, promotes efferocytosis, activates tissue-repair programs, enhances angiogenesis, and selects immunosuppressive TAM populations that drive recurrence (160–164).

Radiotherapy can activate inflammatory macrophage programs through DNA damage, cytosolic DNA sensing, cyclic GMP-AMP synthase-STING (cGAS-STING) activation, and tumor antigen release (164–171). However, radiation can also increase CSF1, CCL2, TGF-β, VEGF, hypoxia, and tissue damage signals, leading to macrophage recruitment, angiogenesis, fibrosis, and immunosuppression. In glioblastoma, ionizing radiation may shift the macrophage compartment from microglia toward infiltrating monocyte-derived macrophages in recurrent tumors, producing a more CSF1- and CCL2-dependent myeloid compartment (147, 172, 173).

Chemotherapy also has dual effects. Certain cytotoxic agents induce ICD and transient macrophage activation, increasing antigen availability and inflammatory signaling. However, repeated or prolonged chemotherapy can promote macrophage-mediated debris clearance, IL-10 and TGF-β production, survival-factor secretion, and tumor-cell repopulation (157, 174, 175). TAM-derived cathepsins, EGF, IGF-1, HGF, WNT ligands, and matrix-remodeling enzymes can protect tumor cells from apoptosis and support the persistence of minimal residual disease (127, 176, 177).

Targeted therapies reshape TAM states by altering tumor-cell cytokine and chemokine output. Tumor cells that escape targeted therapy may rely on macrophage-derived survival factors, including HGF, EGF, IGF-1, and inflammatory cytokines (95, 127, 178). Anti-angiogenic therapy can normalize vasculature and improve immune infiltration in some settings, but excessive vascular pruning may worsen hypoxia and select for HIF-driven angiogenic and suppressive TAM states. PARP inhibitors and DNA-damaging therapies may initially activate innate immunity through cytosolic DNA sensing, but later induce suppressive myeloid programs that limit durable response (178–181).

ICB also remodels TAM compartments. In responding tumors, ICB may be associated with increased inflammatory antigen-presenting myeloid cells and improved macrophage-T-cell crosstalk (28, 114, 182). In resistant tumors, however, TAMs can accumulate as PD-L1-positive, CD73-high, TREM2-positive, SPP1-positive, MARCO-positive, or CD163/HMOX1-associated suppressive populations. These cells impair T-cell priming, exclude effector lymphocytes, promote exhaustion, secrete IL-10 and TGF-β, express inhibitory ligands, and maintain stromal barriers (183, 184).

Therapy-induced TAM plasticity is therefore a major determinant of acquired resistance. The clinical question is not only which TAM states exist at baseline, but also how macrophage states change during treatment and whether these changes create vulnerabilities that can be therapeutically exploited. Accordingly, the TAM categories summarized in **Table 1** should be interpreted as overlapping, context-dependent, and interconverting functional programs rather than mutually exclusive, fixed, or universally conserved macrophage populations; the listed markers are illustrative and may vary with tumor type, anatomical niche, disease stage, and treatment exposure (114, 185–188).

**Table 1:** Overlapping functional states and programs associated with TAMs in cancer.

TAM state/program	Markers/pathway	Function	Clinical relevance
Antigen-presenting TAMs	MHC-II, CD80, CD86, IL-12, STAT1, IRF1	T-cell priming, antigen presentation	May support response to ICB
Immunosuppressive TAMs	IL-10, TGF-β, PD-L1, ARG1, STAT3, STAT6	T-cell suppression, Treg support	Associated with poor ICB response
Angiogenic TAMs	VEGF, TIE2, ANGPT2, MMPs	Abnormal vessel formation, metastasis	Linked to anti-angiogenic resistance
Hypoxic TAMs	HIF-1α, HIF-2α, VEGF, CD73, ARG1	Angiogenesis, metabolic suppression	Enriched in therapy-refractory niches
Lipid-associated TAMs	CD36, APOE, TREM2, FAO pathways	Lipid handling, immune suppression	May promote metabolic resistance
Matrix-remodeling TAMs	MMPs, cathepsins, SPP1, LOX-associated pathways	ECM remodeling, invasion	Associated with metastasis and T-cell exclusion
Efferocytic TAMs	MerTK, AXL, TYRO3, TIM-4	Clearance of apoptotic cells, tissue repair	May drive relapse after cytotoxic therapy

## 3 TAM-mediated cancer resistance and immune evasion

The mechanisms described above converge on a central functional outcome: TAMs create adaptive resistance hubs within tumors. They suppress cytotoxic lymphocytes, impair antigen presentation, express inhibitory checkpoints, block phagocytosis, remodel metabolism, promote angiogenesis, restructure the ECM, communicate via EVs, and support residual tumor-cell survival after therapy. These mechanisms rarely operate independently. Instead, they form integrated resistance networks that differ by tumor type, anatomical site, disease stage, and treatment history (24, 48, 155).

### 3.1 Suppression of T cells, NK cells, antigen presentation, and regulatory immune balance

TAMs suppress antitumor lymphocyte function through soluble mediators, receptor-ligand interactions, metabolic restriction, and spatial exclusion (143, 189). They inhibit CD8-positive T-cell proliferation, cytotoxicity, cytokine production, and memory formation. IL-10, TGF-β, PGE2, and adenosine reduce T-cell receptor signaling, suppress IFN-γ production, and impair effector-cell persistence. Sustained exposure to these signals promotes T-cell exhaustion, characterized by the expression of inhibitory receptors, reduced cytokine production, and epigenetic dysfunction (29, 190).

TAMs also promote T-cell exhaustion through direct checkpoint interactions (29, 191). They express PD-L1, programmed death-ligand 2 (PD-L2), B7 homolog 4 (B7-H4), V-domain immunoglobulin suppressor of T-cell activation (VISTA), galectin-9, and CD80/CD86 in tolerogenic contexts, which engage programmed cell death protein 1 (PD-1), cytotoxic T-lymphocyte-associated protein 4 (CTLA-4), T-cell immunoglobulin and mucin-domain containing-3 (TIM-3), and related receptors. Because TAMs are often positioned at invasive margins, stromal interfaces, and immune-cell clusters, macrophage-derived checkpoint ligands may be functionally important even when tumor-cell PD-L1 is low (191–193).

Antigen presentation is another major point of TAM-mediated immune evasion. Although macrophages can function as antigen-presenting cells (APCs), many TAMs show reduced major histocompatibility complex class II (MHC-II) expression, low costimulation, increased IL-10 signaling, and STAT3- or STAT6-driven suppressive programs (114, 194–196). TAMs can also inhibit dendritic cell (DC) maturation via IL-10, VEGF, PGE2, and TGF-β, thereby weakening T-cell priming in tumor-draining lymph nodes, tertiary lymphoid structures (TLSs), and the tumor itself. Scavenger-like TAM subsets may efficiently capture extracellular material but process antigens in a tolerogenic rather than immunogenic manner (156, 197, 198).

TAMs further suppress immunity by recruiting and supporting Tregs and myeloid-derived suppressor cells (MDSCs) (199). Chemokines such as CCL17, CCL18, and CCL22 attract CCR4-positive Tregs, which suppress effector T cells through IL-10, TGF-β, CTLA-4, and metabolic competition. TAMs and Tregs reinforce one another, creating immunosuppressive niches that correlate with poor CD8-positive T-cell infiltration and unfavorable prognosis. TAM-derived CCL2, C-X-C motif chemokine ligand 8 (CXCL8), IL-6, GM-CSF, VEGF, and PGE2 also recruit and expand monocytic and granulocytic MDSCs, thereby generating a broader myeloid barrier to immune attack (200–202).

Natural killer (NK) cells are also inhibited by TAMs. TGF-β, IL-10, PGE2, adenosine, hypoxia, and lactate reduce NK-cell cytotoxicity, IFN-γ production, and the expression of activating receptors. This is clinically relevant because NK cells contribute to antibody-dependent cellular cytotoxicity (ADCC), meaning TAM-mediated NK suppression may reduce responses to anti-HER2, anti-CD20, anti-EGFR, and other antibody-based therapies (41, 191, 203).

Together, these mechanisms show that TAMs suppress both adaptive and innate antitumor immunity. ICB alone may fail in macrophage-rich tumors if TAMs continue to impair antigen presentation, recruit regulatory populations, suppress NK cells, and physically separate effector lymphocytes from malignant cells (204–206).

### 3.2 Immune checkpoints, Fc receptors, and phagocytosis blockade

TAMs are major non-malignant sources of immune checkpoint ligands and innate inhibitory signals (156). In addition to PD-L1 and PD-L2, TAMs may express B7-H4, VISTA, galectin-9, CD39, CD73, SIRPα, sialic acid-binding immunoglobulin-like lectin (Siglec) receptors, leukocyte immunoglobulin-like receptor B (LILRB) family receptors, and other molecules that suppress T-cell activation or macrophage-mediated phagocytosis (193, 197). Redundancy among these pathways allows tumors to escape single-agent checkpoint blockade. For example, inhibition of PD-1 may be accompanied by compensatory upregulation of VISTA, TIM-3 ligands, B7-H4, adenosine-generating enzymes, or other suppressive pathways within the myeloid compartment (179, 207, 208).

Macrophage phagocytosis is restrained by “don’t-eat-me” signals expressed by tumor cells. The CD47-SIRPα axis is the best-characterized pathway. CD47 on tumor cells binds SIRPα on macrophages and transmits an inhibitory signal that prevents engulfment (7, 29, 209, 210). Other phagocytosis checkpoints include CD24-Siglec-10, MHC-I-LILRB1, and sialic acid-Siglec interactions. These pathways suppress macrophage-mediated tumor-cell clearance and may cooperate with adaptive checkpoints such as PD-1/PD-L1 and CTLA-4 to maintain immune escape (211–213).

The therapeutic effect of phagocytosis checkpoint blockade depends on the balance between pro-phagocytic and anti-phagocytic signals (162, 214). Calreticulin exposure, phosphatidylserine, antibody opsonization, Fc receptor engagement, and macrophage activation promote engulfment, whereas CD47, CD24, MHC-I-associated inhibitory signals, and suppressive TAM states restrain it. Therefore, CD47 or SIRPα blockade may be most effective when combined with tumor-opsonizing antibodies, CD40 agonists, STING or TLR activation, or T-cell checkpoint blockade (28, 212, 215).

Fc-Fc gamma receptor (FcγR) interactions add another layer of resistance. FcγR-bearing TAMs can bind therapeutic antibodies through Fc domains. In some settings, macrophages can remove anti-PD-1 antibodies from T-cell surfaces, reducing receptor occupancy and limiting checkpoint inhibitor efficacy (29, 204, 216, 217). TAMs can also act as antibody sinks or alter antibody-dependent cellular phagocytosis (ADCP) and ADCC depending on antibody isotype, Fc engineering, FcγR expression, and macrophage activation state. Thus, macrophage FcγR profiles can determine whether TAMs enhance or diminish the efficacy of antibody-based therapies (218).

### 3.3 Cytokine, chemokine, and growth-factor networks in resistance

Soluble mediator networks are among the most immediate mechanisms by which TAMs support cancer resistance (219, 220). Tumor cells, CAFs, endothelial cells, lymphocytes, stromal cells, dying cells, and TAMs themselves produce CCL2, CCL5, CXCL12, CSF1, IL-4, IL-6, IL-10, IL-13, TGF-β, VEGF, HGF, IGF-1, EGF, platelet-derived growth factor (PDGF), prostaglandins, and other factors. These mediators recruit myeloid precursors, retain macrophages in tumor niches, and polarize them toward immunosuppressive, pro-angiogenic, tissue-repair, and drug-resistance programs (127, 157, 221, 222).

The CCL2-CCR2 axis promotes monocyte influx, whereas CCL5-CCR5 and CXCL12-CXCR4 signaling support myeloid recruitment and immune exclusion, particularly in stromal-rich tumors. Once established, TAMs secrete chemokines such as CCL17, CCL18, CCL22, and CXCL8 that recruit Tregs, Th2 cells, additional monocytes, and suppressive myeloid populations. This creates feed-forward loops that reinforce a therapy-resistant immune niche (27, 29, 41, 158).

TAM-derived cytokines directly promote tumor-cell survival and immunosuppression. IL-6 activates JAK-STAT3 signaling in tumor cells, increasing antiapoptotic proteins, EMT, stemness, and resistance to chemotherapy or targeted therapy (9, 69, 223–225). IL-10 suppresses antigen presentation and cytotoxic T-cell function while stabilizing suppressive macrophage states. TGF-β promotes fibrosis, immune exclusion, EMT, cancer stemness, and resistance to ICB. TAM-derived IGF-1, HGF, EGF, WNT ligands, growth differentiation factor 15 (GDF15), and PDGF activate PI3K-Akt-mTOR, MAPK, β-catenin, JAK-STAT, and NF-κB pathways in tumor cells, reducing apoptosis and supporting minimal residual disease (95, 226–228).

Proteolytic enzymes released by TAMs further support resistance. Cathepsins, MMPs, heparanases, and related proteases degrade ECM, release matrix-bound growth factors, remodel stromal barriers, and create invasion-permissive tracks. In some models, macrophage-derived cathepsins reduce chemotherapy-induced tumor-cell death, whereas MMPs and other enzymes promote drug resistance, invasion, angiogenesis, and metastasis (133, 229–231).

Macrophage receptor tyrosine kinases (RTKs) integrate several resistance signals (74, 232, 233). Recepteur d’origine nantais/macrophage-stimulating protein receptor (RON/MST1R) promotes PD-L1 expression, suppresses antigen presentation, and enhances T-cell inhibition. TAM-family RTKs, including AXL, Mer tyrosine kinase (MerTK), and TYRO3, promote efferocytosis, immune resolution, suppressive polarization, and inhibition of inflammatory cytokines. These receptors enable tumors to exploit physiological repair pathways to escape immune surveillance and promote post-treatment regrowth (234, 235).

### 3.4 Hypoxia, angiogenesis, metabolism, and vascular resistance

Hypoxia, acidosis, nutrient stress, oxidative stress, and abnormal perfusion are defining features of solid tumors and powerful selectors of therapy-refractory TAM programs (236, 237). Poorly perfused tumor regions activate HIF-1α and HIF-2α in tumor cells and macrophages, inducing VEGF, CXCL12, macrophage migration inhibitory factor (MIF), ARG1, IL-10, PD-L1, matrix-remodeling enzymes, and pro-angiogenic mediators. Hypoxic TAMs frequently acquire angiogenic, immunosuppressive, glycolytic, and tissue-remodeling phenotypes that limit effector T-cell infiltration and reduce sensitivity to chemotherapy, radiotherapy, and immunotherapy (146, 160, 238–240).

Lactate produced by glycolytic tumor cells functions as a signaling molecule rather than simply a metabolic waste product (241–243). Lactate promotes VEGF and ARG1 expression, increases IL-10 and PD-L1, impairs antigen presentation, and reinforces HIF-dependent suppressive macrophage programs. Acidic pH further impairs T-cell and NK-cell function while favoring macrophage states associated with tissue repair, angiogenesis, and immune suppression (244–246).

Nutrient competition contributes to TAM-mediated immune evasion (247). ARG1-expressing TAMs deplete L-arginine, suppressing T-cell proliferation and nitric oxide production. IDO and TDO catabolize tryptophan into kynurenine, promoting T-cell dysfunction, Treg differentiation, and tolerogenic macrophage programming (48, 103, 248). CD39 and CD73 convert extracellular adenosine triphosphate (ATP) released from dying cells into adenosine, which suppresses T cells, NK cells, and myeloid cells through adenosine A2A receptor (A2AR) and adenosine A2B receptor (A2BR) signaling. Lipid-laden TAMs can acquire CD36-dependent lipid uptake, FAO, and ferroptosis-related stress programs that support tumor progression and immunotherapy resistance (95, 249).

Aberrant vasculature intensifies these metabolic pressures. Perivascular TAMs secrete VEGF-A, VEGF-C, ANGPT2, PDGF, CXCL8, MMPs, and other mediators that drive the formation of leaky, tortuous, poorly organized vessels. These vessels create heterogeneous perfusion, elevated interstitial pressure, variable drug delivery, and persistent hypoxia. As a result, cytotoxic drugs may fail to reach tumor cells uniformly, radiotherapy becomes less effective because of oxygen deficiency, and lymphocyte infiltration is restricted (144, 250, 251).

Anti-angiogenic therapy can transiently normalize vasculature and improve drug delivery or immune infiltration, but resistance often emerges. TAMs can restore angiogenesis through non-VEGF pathways, including ANGPT2-TIE2, fibroblast growth factor (FGF), PDGF, MMP-mediated growth-factor release, and hypoxia-driven chemokine secretion. TIE2-positive macrophages are particularly associated with perivascular niches and angiogenic escape. Thus, hypoxia, metabolic stress, angiogenesis, and TAM-mediated suppression form a self-reinforcing loop of resistance (250, 252).

### 3.5 ECM, CAFs, EVs, efferocytosis, and regenerative niches

The desmoplastic stroma is a major driver of TAM-mediated resistance. Dense collagen, fibronectin, hyaluronan, laminins, proteoglycans, and crosslinked ECM networks generate elevated interstitial pressure, tissue stiffness, and complex diffusion paths. These physical barriers reduce penetration of chemotherapeutics, antibodies, nanoparticles, and immune cells. At the same time, ECM architecture directly educates TAMs through integrins, scavenger receptors, discoidin domain receptors (DDRs), mechanosensitive pathways, and cytoskeletal signaling (97, 252, 253).

CAFs and TAMs form reciprocal feed-forward circuits that establish therapy resistance. CAFs secrete CXCL12, IL-6, IL-8, TGF-β, HGF, CCL2, macrophage colony-stimulating factor (M-CSF/CSF1), leukemia inhibitory factor (LIF), periostin, LOX, and ECM components that recruit and polarize macrophages toward immunosuppressive and pro-angiogenic states. TAMs release TGF-β, PDGF, granulin, osteopontin, MMPs, cathepsins, and LOX-associated mediators that activate and maintain CAFs. This mutual reinforcement increases desmoplasia, matrix stiffness, hypoxia, immune exclusion, and drug resistance (139, 140, 254, 255).

TAMs also shape EV-mediated resistance. Tumor-derived EVs educate monocytes and macrophages toward TAM-like states by transferring oncogenic proteins, metabolites, lipids, miRNAs, lncRNAs, and circRNAs (228). These cargos can activate STAT3, PI3K-Akt, NF-κB, HIF-1α, β-catenin, and interferon-related pathways, increasing IL-10, TGF-β, VEGF, PD-L1, and ARG1 while reducing inflammatory cytokine production. TAM-derived EVs reciprocally promote malignant behavior by transferring miRNAs, proteins, and metabolic enzymes to tumor cells, activating EMT, stemness, DNA repair, drug efflux, and survival pathways. EVs released from the tumor site may also condition distant organs, prepare premetastatic niches, and alter circulating immune cells (126, 148, 256).

Efferocytosis links therapy-induced tumor-cell death to immune suppression. After chemotherapy, radiotherapy, targeted therapy, or immune-mediated killing, TAMs engulf apoptotic cells via receptors including MerTK, AXL, TYRO3, TIM-4, stabilin receptors, integrins, and scavenger receptors (257). Although efferocytosis is essential for tissue homeostasis, in tumors it often induces IL-10, TGF-β, prostaglandins, lipid mediators, and tissue repair programs that suppress immunity and promote regrowth. Thus, treatment-induced tumor-cell death can paradoxically educate TAMs toward regenerative and resistance-promoting states (141, 162, 191).

TAMs are also integral components of cancer stem-cell (CSC), dormancy, metastatic, and post-therapy regenerative niches. They interact with stem-like tumor cells via Notch ligands, WNT ligands, activators of the Hedgehog pathway, TGF-β, IL-6, EGF, HGF, IGF-1, osteopontin, gap junctions, and matrix-remodeling enzymes. These signals sustain stemness, quiescence, DNA repair, anti-apoptotic programs, EMT, and resistance to chemotherapy and radiotherapy. Stem-like tumor cells, in turn, secrete CSF1, CCL2, IL-34, periostin, TGF-β, and EV cargos that recruit and polarize TAMs, creating a bidirectional niche that preserves residual disease (149, 162).

At metastatic sites, macrophages and monocytes contribute to premetastatic niche formation through VEGF, VEGF-C, S100 proteins, inflammatory cytokines, EVs, and matrix remodeling (258). Tissue-resident macrophages in liver, lung, bone, brain, and peritoneal niches may either eliminate disseminated tumor cells or, after tumor education, support dormancy, immune evasion, angiogenesis, and outgrowth. Therapy-induced tissue damage further amplifies these regenerative programs, enabling recurrence after initial tumor reduction (61, 259, 260).

Collectively, TAMs coordinate immune suppression, checkpoint signaling, metabolic restriction, angiogenesis, stromal remodeling, extracellular vesicle communication, efferocytosis, and regenerative niche formation to drive cancer immune evasion and therapy resistance (**Figure 2**).

**Figure 2:**
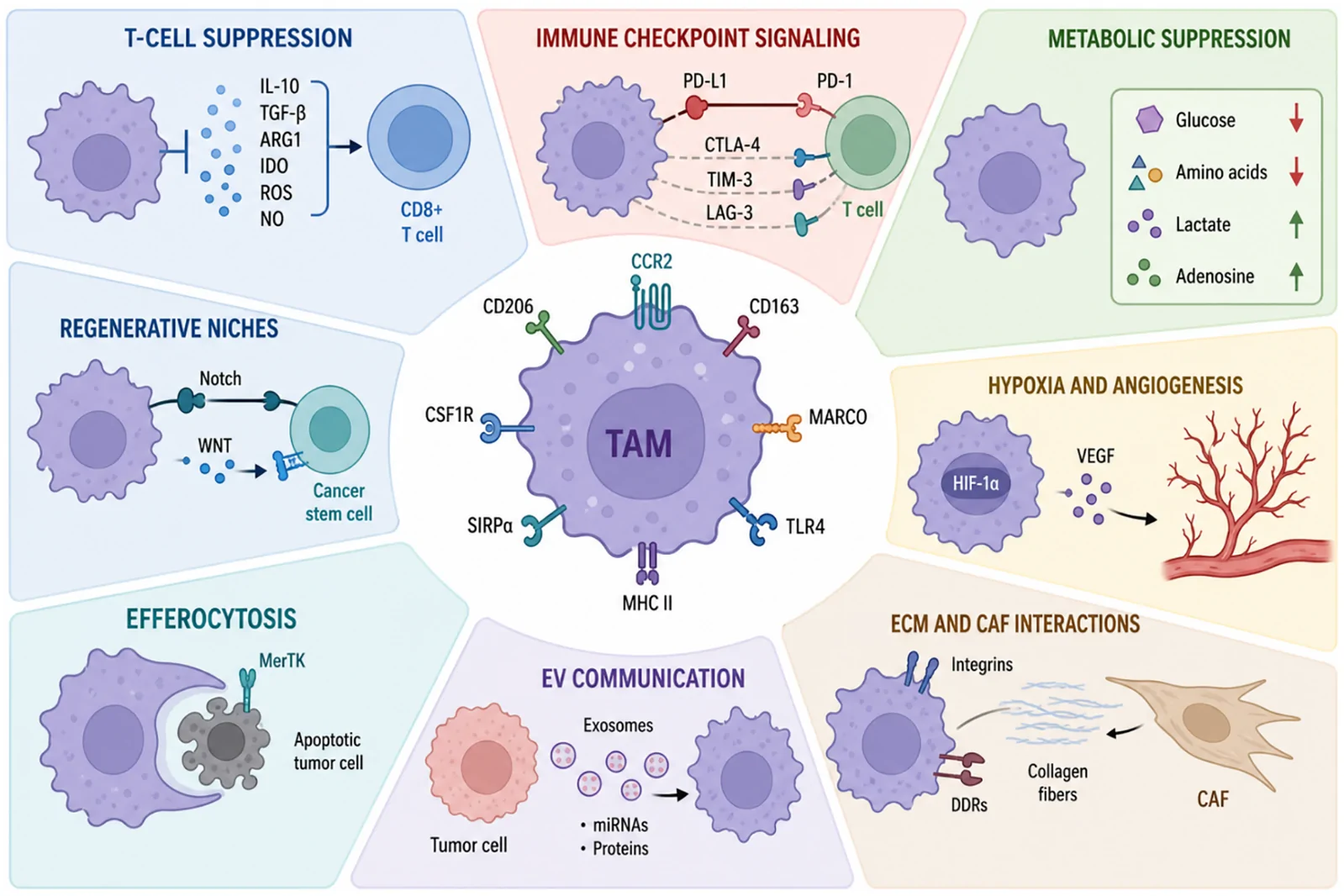
Integrated mechanisms of TAM-mediated immune evasion and cancer therapy resistance. This illustration shows multiple TAM resistance modules, including T-cell suppression, checkpoint signaling, metabolic repression, hypoxia-driven angiogenesis, ECM/CAF crosstalk, EV-based communication, efferocytosis, and regenerative niches. Each module highlights a distinct pathway by which TAMs both inhibit antitumor immunity and reinforce tumor survival, collectively integrating into a coordinated network of immune evasion and therapy resistance.

### 3.6 Integrated view of TAM-driven resistance

TAM-mediated resistance arises from the convergence of immunosuppression, metabolic adaptation, stromal remodeling, angiogenesis, blockade of phagocytosis and efferocytosis, EV communication, and regenerative niche formation. These mechanisms reinforce one another: hypoxia increases adenosine and PD-L1 expression; efferocytosis induces IL-10 and TGF-β; TGF-β promotes ECM deposition and T-cell exclusion; lactate supports angiogenic and suppressive TAM states; and therapy-induced tissue damage recruits macrophages that protect residual tumor cells (127, 261).

Therefore, the central clinical issue is not simply whether TAMs are present, but which macrophage programs dominate, where they are located, how they interact with neighboring cells, and how they change during therapy. This integrated view provides the rationale for therapeutic strategies that deplete, redirect, or functionally disarm pathogenic macrophage states while preserving or enhancing antitumor macrophage functions (**Table 2**) (29, 261–263).

**Table 2:** TAM-mediated mechanisms of resistance and immune evasion.

Mechanism	Key mediators	Resistance effect	Therapeutic prospects
T-cell and NK-cell suppression	IL-10, TGF-β, PGE2, adenosine	Reduced cytotoxicity and cytokine production	ICB plus TAM reprogramming
Impaired antigen presentation	Low MHC-II, low CD80/CD86, IL-10, STAT3	Weak T-cell priming	CD40, STING, TLR agonists
Checkpoint signaling	PD-L1, PD-L2, B7-H4, VISTA, CD73	T-cell exhaustion and ICB resistance	Dual checkpoint or myeloid checkpoint blockade
Phagocytosis blockade	CD47-SIRPα, CD24–Siglec-10, MHC-I–LILRB1	Reduced tumor-cell engulfment	CD47/SIRPα or CD24/Siglec blockade
Hypoxia and angiogenesis	HIF-1α, VEGF, ANGPT2–TIE2	Poor perfusion and immune exclusion	Anti-angiogenic therapy plus TAM targeting
Metabolic suppression	ARG1, IDO/TDO, CD39/CD73, lactate, FAO	Nutrient depletion and lymphocyte dysfunction	Arginase, adenosine, IDO/TDO, lipid pathway inhibitors
ECM and CAF barriers	TGF-β, CXCL12, LOX, MMPs, collagen	Poor drug delivery and T-cell exclusion	Stromal normalization plus immunotherapy
Efferocytosis	MerTK, AXL, TIM-4, phosphatidylserine	Tolerogenic clearance after therapy	MerTK/AXL inhibitors plus immune activation
EV communication	miRNAs, lncRNAs, proteins, metabolites	TAM education and tumor-cell survival	EV biomarkers or engineered EV therapeutics

## 4 Therapeutic reprogramming of TAMs

Therapeutic modulation of TAM biology encompasses interventions with different levels of cellular specificity. Direct TAM-targeted approaches act on receptors or functions enriched in intratumoral macrophages, including CSF1R-dependent survival, selected phagocytosis or efferocytosis checkpoints, and macrophage-directed delivery systems. Broader myeloid-directed approaches, including CCR2 and PI3Kγ inhibition and agonism of CD40, TLR, or STING, can also affect circulating monocytes, dendritic cells, myeloid-derived suppressor cells, and other innate immune populations. Indirect TME-directed strategies, such as vascular or stromal normalization, metabolic intervention, extracellular-vesicle modulation, and microbiome-directed approaches, may alter TAM states by changing the signals and niches that regulate macrophage recruitment and function. These categories overlap, and the biological effect of an intervention depends on target expression, delivery, tumor context, and treatment schedule. Accordingly, “TAM reprogramming” is used here as an umbrella concept, while the cellular specificity of individual approaches is indicated where relevant (9, 76, 98, 263).

Strategies for modulating TAM biology include direct macrophage depletion or reprogramming, broader approaches to myeloid recruitment and activation, restoration of macrophage-mediated phagocytosis, and indirect modification of the stromal, vascular, or metabolic signals that shape TAM function. These interventions may be combined with chemotherapy, radiotherapy, targeted therapy, or immune checkpoint blockade (**Figure 3**).

**Figure 3:**
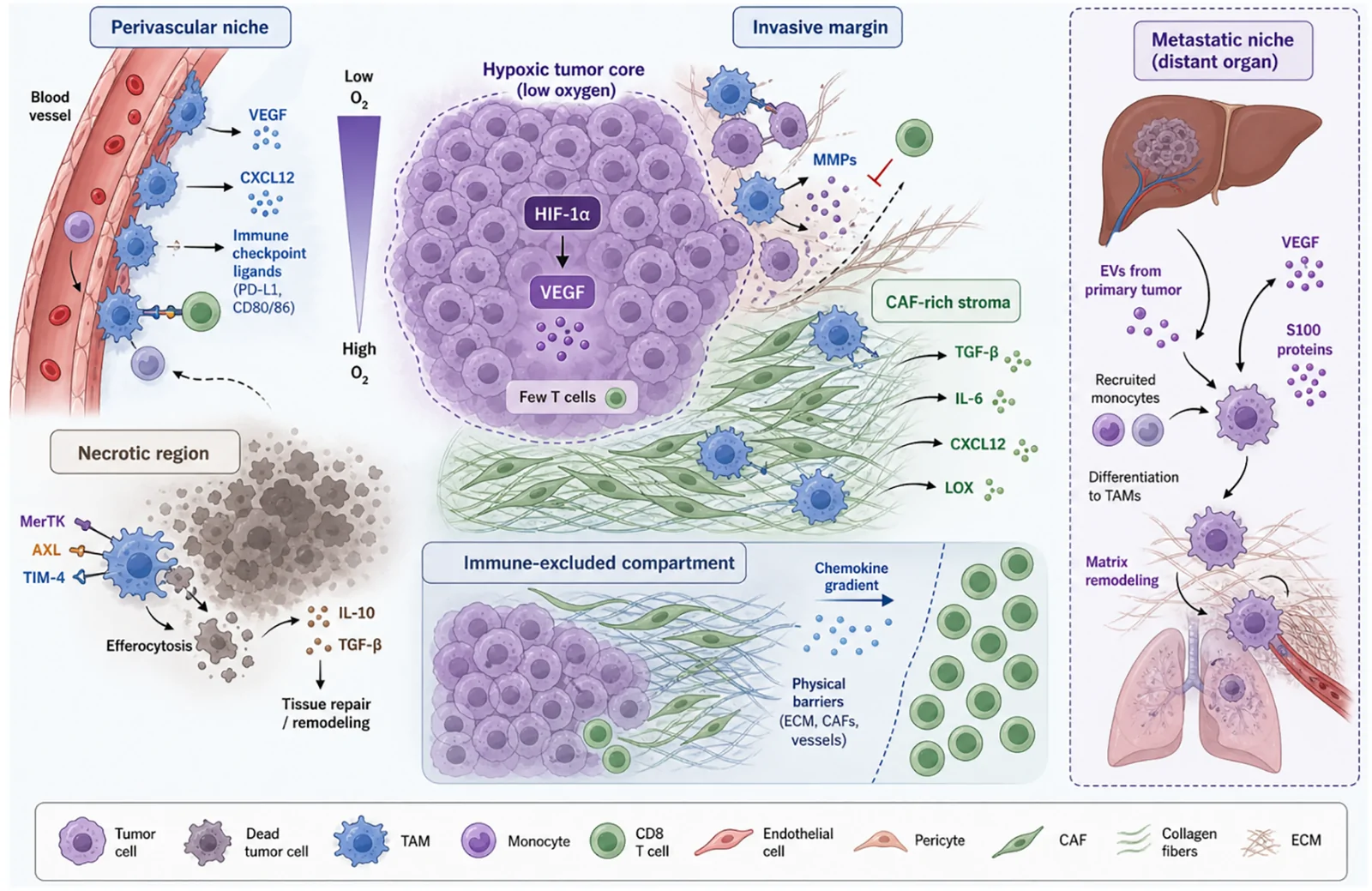
Spatial organization of TAM niches in solid tumors. The illustration shows a cross-section of a solid tumor highlighting the major microanatomical niches in which TAMs accumulate, including hypoxic tumor cores, perivascular areas, invasive margins, CAF-rich stroma, necrotic regions, immune-excluded compartments, and distant metastatic or premetastatic sites. Within each niche, distinct cellular interactions, soluble mediators, oxygen gradients, and extracellular matrix features shape TAM phenotypes and functions, thereby contributing to immune suppression, tumor progression, metastatic dissemination, and resistance to therapy.

The strategies discussed below differ substantially in translational maturity. Established clinical efficacy is currently limited to selected disease contexts, whereas other approaches are supported by early-phase clinical studies, human translational observations, *in vivo* preclinical models, *in vitro* or ex vivo experiments, or remain conceptual and emerging. Evidence level may also vary by tumor type; therefore, clinical validation in a macrophage-dependent disease should not be generalized to heterogeneous solid tumors. **Table 3** indicates the current evidence level for each strategy, and, where available, key clinical claims are supported by original trial reports (9, 26, 28, 29, 264).

**Table 3:** Therapeutic strategies for TAM reprogramming in the TME.

Strategy	Intervention category	Degree of TAM specificity	Current evidence level	Mediator	Mechanism	Limitation	Combinations
TAM depletion	Direct TAM/myeloid targeting	Moderate; CSF1R is also expressed by non-TAM macrophages and monocytes	Established clinical efficacy in CSF1-driven TGCT; limited or early-phase clinical evidence in other tumors	CSF1R inhibitors, anti-CSF1/CSF1R antibodies, liposomal clodronate	Reduces suppressive macrophage burden	Loss of beneficial macrophages; compensatory myeloid influx	ICB, chemotherapy, radiotherapy, microbiome-guided stratification
Recruitment blockade	Broader myeloid modulation	Low-moderate	Early-phase clinical and *in vivo* preclinical evidence	CCR2, CCR5, CXCR4, ANGPT2–TIE2 inhibitors	Limits monocyte recruitment and TAM replenishment	Redundant chemokine pathways; incomplete blockade	CSF1R blockade, ICB, stromal modulation, vascular normalization
Innate activation	Broader innate/myeloid modulation	Low	Early-phase clinical evidence for CD40 agonists; predominantly preclinical evidence for TAM-directed TLR/STING activation	CD40 agonists, TLR agonists, STING agonists	Promotes inflammatory, APC-like TAM states and T-cell priming	Toxicity; tolerance with chronic activation	ICB, vaccines, radiotherapy, oncolytic viruses, nanoparticle delivery
Signaling reprogramming	Broader myeloid or multicellular modulation	Low-moderate	Early-phase clinical evidence for PI3Kγ inhibition; predominantly preclinical evidence for STAT3/STAT6 and HDAC targeting	PI3Kγ, STAT3, STAT6, HDAC inhibitors	Reverses suppressive transcriptional programs and shifts TAMs toward anti-tumor phenotypes	Off-target effects; pathway compensation	ICB, chemotherapy, metabolic inhibitors, epigenetic therapy
Phagocytosis restoration	Direct macrophage-tumor-cell interface targeting	Moderate	Mixed clinical evidence for CD47-SIRPα blockade; predominantly preclinical evidence for other checkpoints	CD47-SIRPα, CD24–Siglec-10, LILRB blockade	Enhances tumor-cell engulfment by macrophages	Toxicity; limited solid tumor penetration	Tumor-opsonizing antibodies, CD40/STING agonists, ICB
Efferocytosis modulation	Direct macrophage/myeloid functional targeting	Moderate	Predominantly *in vivo* preclinical evidence; limited early clinical investigation	MerTK, AXL, TYRO3, TIM-4 inhibitors	Prevents tolerogenic corpse clearance and reduces immunosuppressive macrophage programming	Risk of inflammation/tissue damage	Chemotherapy, radiotherapy, TLR/STING agonists, ICB
Metabolic targeting	Indirect multicellular TME modulation	Low	Target-dependent early clinical evidence; predominantly preclinical evidence for TAM-specific reprogramming	ARG1, IDO/TDO, CD39/CD73, A2AR/A2BR, COX-2, lactate/LDHA, FAO inhibitors	Relieves metabolic immune suppression and disrupts lactate-, adenosine-, arginine-, and tryptophan-driven TAM dysfunction	Redundant metabolism; systemic toxicity; adaptive metabolic rewiring	ICB, TAM reprogramming, epigenetic therapy, stromal normalization
Epigenetic reprogramming	Broad multicellular modulation	Low, unless macrophage-targeted delivery is used	Clinical evidence in cancer broadly; predominantly preclinical evidence for TAM-specific effects	DNMT inhibitors, HDAC inhibitors, BET inhibitors, EZH2 inhibitors	Reverses immune-evasion programs, promotes viral mimicry, and remodels suppressive myeloid states	Broad effects on tumor and normal immune cells	ICB, STING agonists, TAM-targeted therapy, radiotherapy
CAF/TAM crosstalk targeting	Indirect TME-mediated reprogramming	Low	Human translational and *in vivo* preclinical evidence; limited early clinical evidence	FAP-targeted therapy, CXCL12-CXCR4 blockade, IL-6/JAK/STAT3 inhibition, TGF-β inhibition	Disrupts CAF-driven TAM recruitment, immunosuppressive and tissue-repair-associated programming, immune exclusion, and stromal immunosuppression	CAF heterogeneity; risk of removing tumor-restraining CAF subsets	ICB, CSF1R/CCR2 blockade, ECM normalization, vascular normalization
ECM normalization	Indirect TME-mediated reprogramming	Low	Human translational and *in vivo* preclinical evidence; limited clinical validation of TAM-specific benefit	LOX/LOXL2 inhibitors, FAK inhibitors, hyaluronidase, and collagen remodeling approaches	Reduces matrix stiffness, improves immune-cell infiltration, and limits ECM-driven suppressive TAM niches	Risk of enhanced invasion if the ECM is excessively degraded	ICB, nanomedicine, TAM reprogramming, chemotherapy
Vascular normalization	Indirect TME-mediated reprogramming	Low	Established clinical use in cancer; human translational and clinical combination evidence, but indirect TAM-specific effects	Low-dose anti-VEGF therapy, bevacizumab, VEGFR TKIs, ANGPT2 blockade	Reduces hypoxia and abnormal perfusion; limits perivascular TAM-mediated angiogenesis, immune exclusion, and metastasis	Narrow normalization window; excessive inhibition worsens hypoxia	ICB, radiotherapy, chemotherapy, CCR2/CSF1R blockade
Exosome-targeted modulation	Indirect/intercellular signaling modulation	Low or context-dependent	Predominantly *in vitro* and *in vivo* preclinical evidence; conceptual/emerging clinical translation	Inhibitors of exosome biogenesis/release/uptake; exosomal miRNA or PD-L1 targeting	Blocks tumor-derived exosomal education of TAMs toward pro-tumor phenotypes	Delivery and specificity challenges; incomplete understanding of EV cargo	ICB, TAM reprogramming, nanocarriers, liquid biopsy-guided therapy
Delivery platforms	Delivery-enabling strategy	Variable; potentially high with validated targeting	Predominantly *in vivo* preclinical evidence; limited early clinical translation	Nanoparticles, LNPs, engineered EVs, RNA therapy, stimuli-responsive nanocarriers	Improves TAM- or TME-specific delivery; exploits EPR effect, active targeting, acidity, hypoxia, enzymes, or redox cues	Biodistribution, manufacturing, immunotoxicity, and clearance challenges	ICB, vaccines, cytotoxic therapy, STING/TLR agonists, gene editing
Engineered macrophages/myeloid cells	Direct cell-based intervention	High	Early-phase clinical evidence with demonstrated feasibility; efficacy remains unestablished	CAR-macrophages, engineered monocytes, engineered myeloid cells	Direct phagocytosis, antigen presentation, and TME remodeling	Persistence, trafficking, and phenotypic stability	ICB, phagocytosis checkpoint blockade, CD40 agonists, stromal normalization
Microbiome modulation	Indirect systemic modulation	Low	Human translational and early clinical evidence for immunotherapy; indirect TAM-specific evidence remains limited	FMT, defined bacterial consortia, prebiotics, probiotics, and the avoidance of unnecessary antibiotics	Restores pro-immunogenic myeloid tone; supports TAM inflammatory reprogramming and ICI responsiveness	Donor variability, safety, standardization, and regulatory complexity	ICB, CSF1R/CCR2 blockade, CD40 agonists, CD47 blockade
Microbiome-aware trial design	Biomarker/stratification strategy	Not applicable	Human translational evidence; emerging biomarker and trial-design strategy	Baseline stool profiling, antibiotic exposure assessment, responder-associated taxa analysis	Stratifies patients by gut–tumor immune tone and predicts benefit from macrophage-targeted regimens	Biomarker validation and standardization are needed	CSF1R-, CCR2-, CD47-, or CD40-based combinations, ICB
Advanced TAM modeling platforms	Experimental/selection platform	Not applicable	*In vitro*/ex vivo and human translational evidence; emerging research and selection platform	Patient-derived organoids with myeloid co-culture, tumor-on-a-chip, 3D bioprinting, scRNA-seq, spatial omics, AI profiling	Models TAM-tumor-CAF-vessel interactions and identifies patient-specific resistance signatures	Technical complexity; limited clinical standardization	Functional drug screening, biomarker discovery, adaptive trial design
Integrated TME reprogramming	Multicellular ecosystem modulation	Low	Conceptual framework supported by preclinical and selected clinical combination evidence	Combined TAM targeting with stromal, vascular, metabolic, epigenetic, and microbiome interventions	Converts macrophage-rich “cold” or immune-excluded tumors into more inflamed, therapy-responsive TMEs	Complex dosing, sequencing, toxicity, and trial design	ICB, radiotherapy, nanomedicine, microbiome modulation, adaptive monitoring

TAM specificity is relative rather than absolute. “High” indicates direct macrophage use or targeting; “moderate” indicates macrophage-relevant targets shared with other cells; and “low” indicates broader myeloid, stromal, vascular, metabolic, or systemic effects. Evidence levels are tumor- and strategy-dependent: “established” denotes randomized clinical evidence; “early clinical” denotes preliminary phase I/II evidence; “human translational” denotes patient-derived evidence; and “preclinical” denotes *in vivo*, *in vitro*, or ex vivo evidence. Clinical validation of a strategy does not necessarily establish a TAM-specific benefit.

### 4.1 TAM depletion and recruitment blockade

Macrophage depletion is one of the earliest strategies for reducing myeloid-mediated immune suppression. This approach is based on the observation that many tumors are enriched with TAM populations that promote angiogenesis, matrix remodeling, T-cell exclusion, therapy resistance, and metastasis. Depleting these cells can reduce the immunosuppressive burden and create a more permissive environment for antitumor immunity (8, 28).

The most widely studied depletion strategy targets CSF1R, which is required for macrophage differentiation, survival, and tissue maintenance (29). Small-molecule CSF1R inhibitors, including pexidartinib and related agents, as well as monoclonal antibodies against CSF1 or CSF1R, can reduce TAM density or alter TAM phenotype in preclinical models and selected clinical settings. CSF1R inhibition may also enhance chemotherapy, radiotherapy, anti-angiogenic therapy, or ICB by limiting macrophage-mediated repair and immune suppression (265, 266).

Other depletion strategies include liposomal clodronate, which is phagocytosed by macrophages and induces apoptosis, and trabectedin, which can affect mononuclear phagocytes in certain tumor contexts. However, broad depletion is biologically complex because not all macrophages are harmful. Some macrophage subsets contribute to antigen presentation, tumor-cell phagocytosis, and tissue homeostasis. Broad depletion may therefore remove beneficial macrophages along with suppressive populations (2, 263, 267).

An alternative approach is to block monocyte recruitment into tumors. CCR2 inhibition can reduce CCL2-mediated monocyte recruitment, decrease TAM accumulation, and improve responses to chemotherapy or immunotherapy in preclinical models (268). Additional recruitment pathways include CCL5-CCR5, CXCL12-CXCR4, VEGF-VEGFR, CSF1-CSF1R, and ANGPT2-TIE2. Because these pathways are redundant, blockade of a single axis may be bypassed by alternative chemokine or growth-factor networks. Dual or sequential targeting, such as CCR2 plus CXCR4 or CCR2 plus CSF1R inhibition, may be required in selected tumors (269, 270).

The main limitation of depletion or recruitment blockade is that macrophage compartments can rapidly adapt (21). Incomplete depletion, compensatory granulocytic myeloid expansion, rebound monocyte mobilization, and the survival of tissue-resident macrophages may limit the durability of benefit. Therefore, depletion and recruitment blockade are most likely to succeed in biomarker-defined tumors that are strongly dependent on suppressive monocyte-derived TAMs and should be combined with therapies that activate adaptive immunity or prevent compensatory myeloid escape (29, 190).

### 4.2 Reprogramming TAMs toward antitumor immunity

TAM reprogramming aims to convert macrophages from tumor-supportive states into inflammatory, antigen-presenting, phagocytic, and T-cell-stimulating cells. Reprogrammed TAMs can increase MHC and costimulatory molecule expression, produce IL-12, TNF, and IFN-γ-associated chemokines, enhance tumor-cell phagocytosis, and support cytotoxic T-cell recruitment (61, 127).

CD40 agonists are among the best-developed myeloid-activating strategies. CD40 activation promotes the immunostimulatory differentiation of macrophages, DCs, and B cells. In TAMs, CD40 agonism can enhance antigen presentation, inflammatory cytokine production, tumor-cell phagocytosis, and T-cell priming (29, 157). CD40 agonists are particularly attractive in tumors with poor DC function or dense myeloid infiltration, such as PDAC. However, systemic CD40 activation can cause cytokine-release-related toxicity, liver enzyme elevation, thrombocytopenia, and inflammatory adverse events, requiring careful dose, formulation, and route optimization (271).

TLR agonists can activate macrophages through NF-κB and interferon regulatory factor pathways. TLR7/8 and TLR9 agonists can stimulate inflammatory cytokine production, antigen presentation, and effector-cell recruitment. Intratumoral or tumor-targeted TLR delivery may convert immunologically cold tumors into inflamed lesions while limiting systemic toxicity. However, chronic or poorly timed TLR activation may induce IL-10 and PD-L1 expression, suppressive myeloid expansion, or macrophage tolerance (272, 273).

STING pathway activation is another promising strategy. cGAS-STING signaling senses cytosolic DNA and induces IFN-I responses, antigen presentation, and CXCL9/CXCL10-mediated T-cell recruitment. STING agonists may be particularly useful in tumors with low baseline interferon signaling or poor T-cell infiltration. Yet excessive or chronic STING activation can promote tissue damage, immune exhaustion, or compensatory checkpoint expression, and delivery remains a major challenge (91, 168).

PI3Kγ inhibition reprograms suppressive myeloid cells by targeting a central node of macrophage signaling (274). PI3Kγ promotes immunosuppressive transcriptional programs while limiting inflammatory activation. Inhibitors such as eganelisib have been explored with PD-1 or PD-L1 blockade and other therapies. Unlike broad depletion, PI3Kγ inhibition aims to preserve macrophages while changing their function. Clinical benefit may depend on selecting patients with macrophage-rich, myeloid-inflamed, or checkpoint-resistant tumors (275).

Intracellular pathways such as STAT3, STAT6, NF-κB, mTOR, JAK, MAPK, Bruton tyrosine kinase (BTK), spleen tyrosine kinase (SYK), and HIF signaling also regulate TAM phenotype. STAT3 inhibition may reduce IL-10, PD-L1, and VEGF, and suppress the production of suppressive cytokines. STAT6 inhibition may limit IL-4/IL-13-driven tissue repair and fibrosis-associated immune exclusion. NF-κB modulation requires caution because controlled activation can promote tumoricidal inflammation, whereas complete inhibition may impair beneficial immunity (190, 276, 277).

Epigenetic reprogramming offers another route to durable conversion of the macrophage state. HDAC inhibitors, DNMT inhibitors, BET inhibitors, and histone methylation modulators can alter macrophage chromatin accessibility, inflammatory responses, antigen presentation, and cytokine production. However, epigenetic agents affect multiple cell types, including tumor cells, T cells, DCs, CAFs, and endothelial cells. Future development should emphasize macrophage-targeted delivery and pharmacodynamic confirmation of TAM state conversion (89, 114, 115).

### 4.3 Restoring phagocytosis and modulating efferocytosis

Restoring macrophage phagocytosis is a major therapeutic goal. Tumor cells evade engulfment by expressing inhibitory signals such as CD47, CD24, MHC-I, and sialylated glycans. CD47-SIRPα blockade is the most advanced approach (278, 279). Anti-CD47 antibodies, SIRPα fusion proteins, and engineered molecules can restore macrophage-mediated tumor-cell engulfment and may enhance ADCP. Other targets include CD24-Siglec-10, MHC-I-LILRB1, PD-1/PD-L1 effects on macrophage phagocytosis, and sialic acid-Siglec interactions (212).

Phagocytosis checkpoint blockade may be especially effective when combined with tumor-opsonizing antibodies such as anti-CD20, anti-HER2, or anti-EGFR agents (28). However, phagocytosis alone may not be sufficient for durable immunity. Engulfed tumor material must be processed in a way that supports antigen presentation and T-cell priming rather than silent clearance. Therefore, phagocytosis checkpoint blockade may need to be paired with CD40 agonists, STING agonists, TLR agonists, checkpoint inhibitors, or vaccines (280).

Efferocytosis pathways are also therapeutic targets. After chemotherapy or radiotherapy, TAMs engulf apoptotic tumor cells through MerTK, AXL, TYRO3, TIM-4, stabilin receptors, and phosphatidylserine-recognition pathways. This often induces IL-10, TGF-β, pro-resolving lipid mediators, and tissue-repair programs that suppress immunity and support recurrence. Inhibiting MerTK, AXL, TYRO3, TIM-4, or related pathways may prevent macrophages from converting tumor-cell death into immune tolerance (151, 162).

Because efferocytosis is essential for normal tissue homeostasis, systemic inhibition may cause inflammation, autoimmunity, or tissue damage. Complete blockade of apoptotic-cell clearance may also worsen necrosis. Therefore, modulation of efferocytosis should be localized, time-limited, or combined with immune-activating agents that redirect corpse clearance toward immunogenic antigen presentation (261, 281).

Additional TAM surface receptors may provide more selective targets. TREM2-positive TAMs have been associated with immunosuppression and checkpoint resistance across multiple tumor types (195, 282). MARCO-targeting strategies may reprogram scavenger-like macrophages. Clever-1/stabilin-1 blockade may convert suppressive macrophages into inflammatory APCs. LILRB and Siglec pathways may suppress both phagocytosis and inflammatory activation. These targets require companion diagnostics to confirm expression on pathogenic intratumoral macrophage subsets and to determine whether the receptor is a functional driver rather than merely a marker (283, 284).

### 4.4 Targeting TAM metabolism, cytokine networks, hypoxia, and stromal barriers

TAM function is tightly linked to metabolism, making metabolic reprogramming an attractive therapeutic strategy. Arginase inhibitors can preserve arginine availability for T-cell activation and reduce myeloid-mediated nutrient deprivation (196, 285). IDO and TDO inhibitors target tryptophan catabolism and kynurenine production, although clinical experience with IDO inhibition suggests that blocking a single metabolic enzyme may be insufficient without appropriate patient selection and combination therapy. Adenosine-axis inhibitors, including antagonists of CD39, CD73, A2AR, and A2BR, aim to reverse purinergic immune suppression. Cyclooxygenase-2 (COX-2)/PGE2 blockade may improve T-cell and NK-cell function and reduce suppressive macrophage activity (162, 191, 286).

Lactate and lipid metabolism are emerging targets. Tumor-derived lactate promotes angiogenic and immunosuppressive TAM programs, while lipid accumulation supports oxidative and repair-like macrophage states. FAO inhibitors, lactate transport inhibitors, lipid uptake modulators, and iron-handling interventions may alter TAM phenotype and sensitize tumors to therapy. Iron oxide nanoparticles, including ferumoxytol, may have diagnostic and immunomodulatory effects by influencing macrophage polarization while enabling imaging (108, 287).

Modulation of the cytokine and chemokine network can indirectly reprogram TAMs and reshape the TME. TGF-β blockade is particularly relevant in immune-excluded tumors, where it promotes fibroblast activation, ECM deposition, T-cell exclusion, and the development of suppressive macrophage states. IL-6-STAT3 inhibition may reduce tumor-promoting inflammation and myeloid suppression (102). IL-1β blockade may limit inflammasome-driven myeloid recruitment and angiogenesis. IL-10 pathway modulation may restore antigen presentation and inflammatory cytokine production. Chemokine inhibitors targeting CCR2, CCR5, C-X-C motif chemokine receptor 2 (CXCR2), or CXCR4 may reduce the recruitment of suppressive myeloid cells and improve immune-cell positioning (204, 288).

Hypoxic and angiogenic TAM programs can be targeted through vascular normalization and hypoxia-directed therapy. Anti-VEGF and VEGFR inhibitors can transiently normalize abnormal vessels and improve immune infiltration. ANGPT2-TIE2 targeting may disrupt proangiogenic macrophage programs. HIF inhibitors may reduce hypoxia-induced macrophage polarization, adenosine production, and VEGF expression. However, excessive vascular pruning may worsen hypoxia and reinforce suppressive TAM states, making dose and timing critical (289).

Stromal barriers can be addressed through TGF-β inhibition, CXCR4 blockade, LOX inhibition, FAK inhibition, DDR inhibition, integrin antagonists, hyaluronidase, focal radiotherapy, and other matrix-modifying strategies. The goal should be stromal normalization rather than indiscriminate matrix destruction, because some fibroblast and ECM components may restrain tumor spread. Combining stromal modulation with TAM reprogramming and T-cell activation may convert immune-excluded tumors into immune-infiltrated tumors (252).

### 4.5 Nanoparticles, RNA-based therapy, EVs, and engineered macrophages

Nanoparticle platforms offer a promising means of delivering TAM-reprogramming agents directly to macrophages while reducing systemic toxicity. Macrophages naturally phagocytose particulate materials, enabling the engineering of liposomes, polymeric nanoparticles, lipid nanoparticles (LNPs), albumin-based particles, dendrimers, iron oxide nanoparticles, and biomimetic vesicles for TAM uptake. Surface modification with mannose, antibodies, peptides, or scavenger receptor ligands can enhance the targeting of specific macrophage subsets (157, 290).

Nanoparticles can carry small-molecule inhibitors, nucleic acids, cytokines, TLR or STING agonists, metabolic modulators, epigenetic agents, or chemotherapeutics. Iron oxide nanoparticles are especially interesting because they may serve both imaging and immunomodulatory functions. However, clinical translation remains limited by heterogeneity in biodistribution, tumor penetration, manufacturing complexity, safety concerns, and uncertainty about which TAM subsets are targeted in human patients. Companion diagnostics should confirm intratumoral accumulation and macrophage uptake (291–293).

RNA-based therapies provide programmable tools for altering TAM function. Small interfering RNA (siRNA), antisense oligonucleotides (ASOs), miRNA mimics, miRNA inhibitors, and messenger RNA (mRNA) delivery systems can suppress protumor genes or induce antitumor programs (157). Potential targets include STAT3, HIF-1α, VEGF, CSF1R, CCR2, PI3Kγ, ARG1, IL-10, CD206, and other genes involved in immune suppression. Conversely, mRNA delivery could express inflammatory cytokines, costimulatory molecules, or transcription factors that promote antigen presentation and tumoricidal activity. The major challenge is the selective delivery of RNA to intratumoral TAMs without excessive uptake in the liver or spleen (28, 294).

EV-based strategies can be used either to block protumor vesicle signaling or to engineer vesicles as delivery vehicles (29). Tumor-derived EVs educate macrophages toward suppressive states by transferring oncogenic proteins, metabolites, lipids, and non-coding RNAs. Inhibiting EV release, uptake, or cargo function may reduce tumor-induced TAM polarization. Conversely, engineered EVs could deliver miRNAs, siRNAs, proteins, or immune-stimulatory molecules to macrophages. Standardization of EV isolation, cell-of-origin assignment, potency, biodistribution, and safety remains essential (295, 296).

Engineered macrophage and CAR-macrophage therapies represent an emerging cellular approach. CAR-macrophages are designed to recognize tumor antigens, phagocytose cancer cells, remodel the TME, and stimulate T-cell responses through antigen presentation. Compared with CAR-T cells, macrophages may be better suited to infiltrate dense solid tumors and survive in hostile microenvironments (297, 298). Engineered macrophages may also be programmed to express cytokines, enzymes, costimulatory molecules, or gene-editing components. Key challenges include manufacturing consistency, persistence, trafficking, phenotypic stability, safety, and the prevention of conversion to suppressive macrophages upon entering the TME (299).

### 4.6 Combination strategies with immunotherapy, cytotoxic therapy, vaccines, and microbiome modulation

TAM-targeted therapies are unlikely to achieve durable responses as monotherapies in most solid tumors. The most promising strategies will combine macrophage modulation with treatments that release tumor antigens, activate T cells, normalize vasculature, remodel stroma, or enhance innate immune recognition (61).

Combining TAM-targeted therapy with ICB is one of the most clinically important strategies. TAMs suppress checkpoint therapy by excluding T cells, expressing inhibitory ligands, producing suppressive cytokines, limiting antigen presentation, and generating adenosine-rich niches (38). Potential combinations include PD-1 or PD-L1 inhibitors with CSF1R inhibitors, CCR2 inhibitors, PI3Kγ inhibitors, CD40 agonists, STING agonists, TLR agonists, adenosine-axis inhibitors, anti-angiogenic therapy, or stromal normalization agents. Patient selection should focus on macrophage-rich, myeloid-inflamed, immune-excluded, or checkpoint-resistant tumors (114).

Chemotherapy and radiotherapy can increase tumor antigen release and induce ICD, but they can also recruit suppressive macrophages that clear dying cells, promote tissue repair, and support relapse (28). TAM-targeted therapy may improve the immune consequences of cytotoxic treatment. CSF1R, CCR2, MerTK, AXL, and PI3Kγ inhibitors may prevent therapy-induced macrophage-mediated resistance, while CD40, STING, or TLR agonists may convert treatment-induced tumor-cell death into productive antigen presentation. Timing is critical: macrophage reprogramming may be most effective after antigen release but before suppressive efferocytosis and tissue-repair programs dominate (151).

Oncolytic viruses and cancer vaccines can also be paired with TAM reprogramming. Oncolytic viruses induce tumor-cell lysis, release antigens, and activate innate immune sensing. Vaccines improve tumor-specific T-cell priming but may fail if TAMs suppress antigen presentation or exclude effector cells. CD40, STING, TLR, CSF1R, or PI3Kγ modulation may enhance vaccine- or virus-induced immunity by converting macrophages into APCs that support T-cell responses (28, 151).

Anti-angiogenic and stromal-targeting therapies can improve immune-cell access and drug delivery when used appropriately. Vascular normalization may enhance lymphocyte infiltration and reduce hypoxia-driven suppression of TAMs. ECM and CAF modulation may relieve immune exclusion and improve delivery of antibodies, nanoparticles, and adoptive cells. These approaches should be sequenced with TAM reprogramming and ICB to maximize immune engagement (206, 300).

The microbiome is an emerging modulator of TAM function and immunotherapy response (250). Microbial metabolites, PRR ligands, and antibiotic-induced dysbiosis can alter macrophage development, inflammatory tone, and checkpoint responsiveness. Microbiome-based strategies, including dietary modulation, probiotics, prebiotics, fecal microbiota transplantation, microbial metabolite supplementation, and avoidance of unnecessary antibiotics, may indirectly improve TAM function. However, microbiome effects are variable and require prospective validation with integrated myeloid immune profiling (264, 301).

Personalized TAM-directed therapy should match the intervention to the dominant macrophage program. Tumors dominated by suppressive macrophage accumulation may benefit from depletion; tumors with continuous monocyte influx may require recruitment blockade; tumors with phagocytosis blockade may need CD47/SIRPα or related checkpoint targeting; metabolically suppressive tumors may require adenosine, arginine, tryptophan, lactate, or lipid pathway modulation; and immune-excluded tumors may require TAM reprogramming plus stromal or vascular normalization (29, 141, 302).

In summary, the major therapeutic strategies for TAM reprogramming include inhibition of macrophage recruitment and survival; activation of CD40, TLR, or STING signaling; restoration of phagocytosis; modulation of efferocytosis; metabolic and epigenetic targeting; stromal and vascular normalization; nanoparticle-based delivery; and engineered-macrophage therapies. When combined with immune checkpoint blockade, chemotherapy, radiotherapy, vaccines, anti-angiogenic therapy, or microbiome-guided approaches, these strategies can redirect TAMs from tumor-supportive states toward inflammatory, phagocytic, and T-cell-supportive antitumor functions (**Figure 4**).

**Figure 4:**
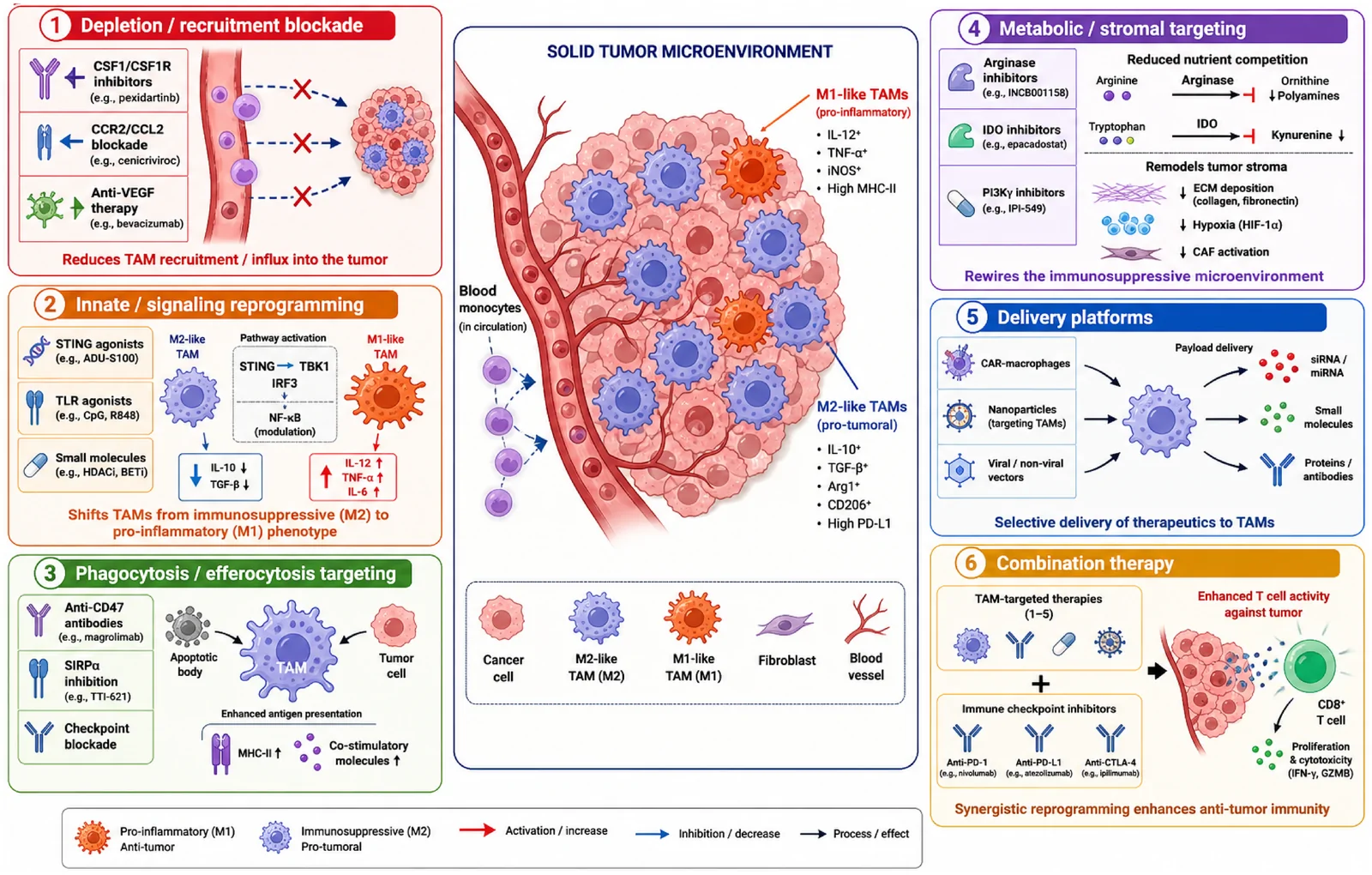
Therapeutic reprogramming strategies targeting TAMs. The illustration outlines major therapeutic strategies for modulating TAMs within the TME, blocking macrophage recruitment or survival, reprogramming innate immune signaling, targeting phagocytosis and efferocytosis pathways. Subsequently, disrupting metabolic and stromal support mechanisms, using specialized delivery platforms, and combining TAM-directed approaches with conventional therapies or immune checkpoint blockade. Centered around a tumor model, the figure demonstrates how interventions such as CSF1/CSF1R or CCL2/CCR2 inhibition, STING and TLR agonists, CD47-SIRPα blockade, metabolic inhibitors, CAR-macrophage therapy, and checkpoint inhibitors can redirect TAMs away from immunosuppressive, tumor-supportive functions toward a more inflammatory, tumoricidal state that strengthens T cell-mediated antitumor immunity.

## 5 Ecosystem-level integration of TAM programming and therapeutic response

The mechanisms described in Sections 2–4 operate as an interconnected TME ecosystem rather than as independent pathways. Tumor cells, CAFs, ECM, immune populations, metabolic gradients, extracellular vesicles, and systemic microbiome-derived signals collectively regulate TAM recruitment, localization, functional state, and treatment response. For example, CAF-derived cytokines and matrix deposition promote myeloid recruitment and immune exclusion; abnormal vasculature intensifies hypoxia and metabolic stress; and hypoxia, lactate, adenosine, and apoptotic cell clearance reinforce angiogenic, efferocytic, and immunosuppressive macrophage programs. TAMs reciprocally sustain this ecosystem by promoting vascular remodeling, fibroblast activation, matrix turnover, regulatory immune-cell recruitment, and tumor-cell survival (94, 150, 228, 303, 304).

This interdependence helps explain why isolated macrophage-directed interventions often produce incomplete or transient effects. Direct TAM targeting may need to be combined with broader myeloid modulation or indirect TME-directed strategies, including vascular normalization, stromal remodeling, metabolic intervention, antigen-releasing therapy, or immune checkpoint blockade. The appropriate combination should be determined by the dominant resistance architecture rather than by TAM abundance alone. Effective treatment therefore requires evidence that the targeted TAM program is functionally active, spatially accessible, and linked to therapeutic resistance in the relevant tumor (23, 155, 305–309).

Human-relevant experimental platforms may support this mechanism-guided approach. Patient-derived organoids with autologous immune or stromal co-culture, ex vivo tumor slices, tumor-on-a-chip systems, three-dimensional bioprinting, and single-cell and spatial multi-omics can model TAM–tumor–stroma interactions, spatial barriers, and treatment-induced macrophage-state changes (20, 97, 303, 304, 310, 311). These platforms remain complementary research tools rather than validated clinical decision systems, but their integration with longitudinal patient sampling and artificial intelligence-guided analysis may improve biomarker development, functional drug testing, and the prioritization of rational combinations.

Thus, ecosystem-level TAM reprogramming should be viewed as a mechanism-guided framework rather than a single therapeutic class. Its clinical value will depend on matching direct TAM-targeted, broader myeloid-directed, or indirect TME-mediated interventions to the dominant cellular and spatial resistance program and confirming that treatment produces durable functional reprogramming rather than transient target engagement. The principal ecosystem-level strategies and their relative TAM specificity are summarized in **Table 3**; **Figure 5**.

**Figure 5:**
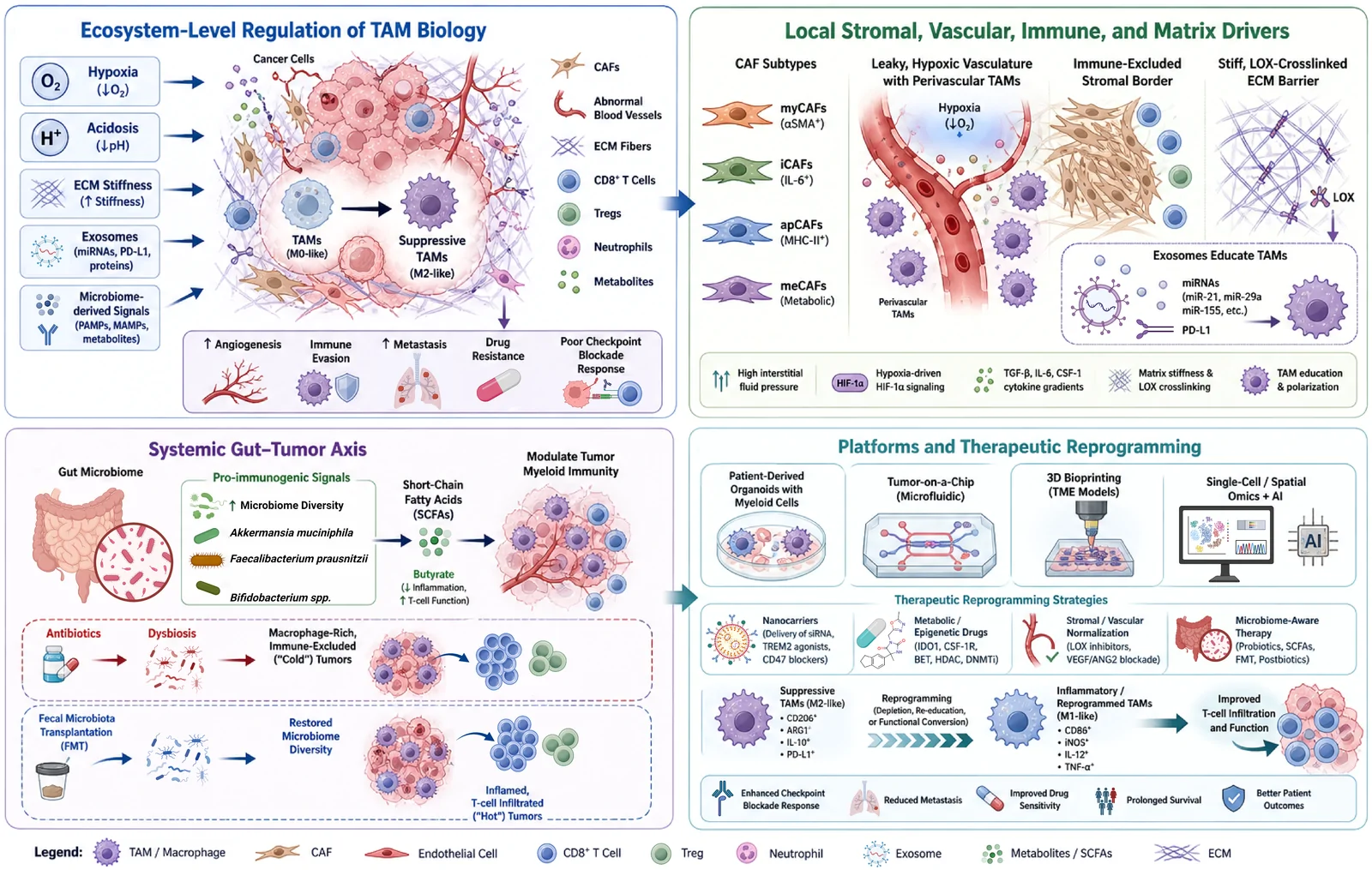
Tumor microenvironment ecosystem control of TAM biology, modeling, and therapeutic reprogramming. This illustration shows how the broader tumor ecosystem shapes TAMs through CAFs, abnormal vessels, ECM stiffness, hypoxia, microbiome signals, and tumor-derived exosomes. It also highlights experimental models and therapies that can reprogram suppressive TAMs into more inflammatory, antitumor states.

## 6 Clinical translation and future directions

The clinical translation of TAM-targeted therapy requires a shift from measuring macrophage abundance alone toward defining macrophage state, origin, spatial localization, receptor expression, metabolic program, and therapy-induced adaptation. TAMs are not a uniform therapeutic target; they comprise diverse populations with distinct ontogenies, functional programs, and niche-specific activities (24). Some TAM subsets suppress T-cell and NK-cell function, promote angiogenesis, remodel the extracellular matrix, mediate efferocytosis, and support relapse, whereas others may contribute to antigen presentation, tumor-cell phagocytosis, and immune activation. Therefore, future clinical strategies must distinguish pathogenic macrophage states from beneficial or potentially reprogrammable populations before selecting depletion, recruitment blockade, phagocytosis restoration, metabolic inhibition, or immune-activating approaches.

A major limitation of current clinical trials is insufficient patient stratification according to TAM biology. TAM composition varies across cancer types, patients, metastatic sites, and spatial regions within the same lesion, where tissue-resident and monocyte-derived macrophages may coexist with antigen-presenting, hypoxic, angiogenic, efferocytic, lipid-associated, matrix-remodeling, TREM2-positive, SPP1-positive, and other immunosuppressive or inflammatory states (21, 27, 29, 62, 132). These populations may differ in receptor expression, metabolic dependence, localization, and sensitivity to therapy. Consequently, a single-target intervention may eliminate or reprogram one TAM population while sparing spatially inaccessible or molecularly distinct subsets. Inhibition of one recruitment, survival, or suppressive pathway may also promote alternative myeloid recruitment or adaptive state transitions, allowing therapeutic escape. Agents targeting CSF1R, CCR2, PI3Kγ, CD40, STING, CD47-SIRPα, adenosine, arginase, IDO/TDO, or epigenetic regulators are nevertheless frequently evaluated in heterogeneous patient populations without confirming that the targeted pathway is dominant or broadly expressed within the tumor. Baseline profiling should therefore define TAM ontogeny, functional state, spatial distribution, receptor expression, and metabolic program to identify patients likely to benefit and to determine whether simultaneous or sequential targeting of multiple macrophage programs is required (21, 27, 29, 62, 132).

Advanced technologies are central to this precision approach. Single-cell RNA sequencing, spatial transcriptomics, multiplex immunofluorescence, imaging mass cytometry, spatial proteomics, metabolomics, phosphoproteomics, and digital pathology can define TAM heterogeneity with increasing resolution (9). These tools can distinguish recruited monocyte-derived macrophages from tissue-resident populations, map TAM localization in hypoxic cores, perivascular regions, invasive margins, CAF-rich stroma, immune-excluded compartments, and metastatic niches, and identify ligand-receptor interactions between TAMs, tumor cells, CAFs, endothelial cells, T cells, and dendritic cells. Spatial biomarkers are particularly important because bulk markers such as CD68, CD163, or CD206 may obscure functionally distinct macrophage programs and fail to predict therapeutic response.

Longitudinal monitoring will also be essential because TAM phenotype and metabolism can change rapidly during tumor evolution and treatment. Fluctuations in hypoxia, acidity, lactate, lipids, nutrient availability, cytokines, extracellular matrix architecture, and therapy-induced damage can redirect macrophages among inflammatory, angiogenic, efferocytic, tissue-repair, and immunosuppressive states (21, 29, 97, 133, 312). Consequently, a single intervention may produce an initial TAM-state conversion without maintaining durable efficacy, as residual macrophages can use alternative signaling or metabolic pathways and reacquire tumor-supportive functions. Chemotherapy, radiotherapy, targeted therapy, anti-angiogenic therapy, immune checkpoint blockade, oncolytic virotherapy, and adoptive cell therapy may further select for or induce macrophage populations that differ from those present at baseline. A pretreatment biopsy may therefore fail to capture therapy-induced efferocytic, angiogenic, wound-healing, or PD-L1/CD73-high suppressive TAM states that drive acquired resistance. Serial tumor biopsies, circulating monocyte profiling, extracellular-vesicle cargo analysis, cytokine monitoring, hypoxia or metabolic imaging, immunoPET, and spatial immune mapping should be incorporated into clinical studies when feasible. These approaches can determine whether treatment produces sustained inflammatory and antigen-presenting macrophage states or instead induces adaptive programs that support residual disease and relapse (21, 26, 29, 132).

The design of TAM-directed combination therapy presents additional clinical challenges. Combining TAM reprogramming with ICB, chemotherapy, radiotherapy, targeted therapy, or anti-angiogenic therapy may overcome complementary resistance mechanisms but can also lead to overlapping toxicities, pharmacodynamic antagonism, or excessive immune activation. The optimal dose, treatment duration, sequence, and timing depend on the dominant macrophage program and the biological effects of the partner therapy (25, 26, 29, 132). For example, blockade of monocyte recruitment may be required before or during cytotoxic treatment to prevent therapy-induced macrophage accumulation, whereas macrophage activation may be more effective after antigen release but before tolerogenic efferocytosis and tissue-repair programs become dominant. ICB may benefit from prior vascular normalization, ECM remodeling, or inhibition of CXCR4 or TGF-β to improve lymphocyte access to tumor nests. Simultaneous administration may not always be preferable, because excessive pathway inhibition or innate activation could narrow the safety window without improving efficacy. Early-phase trials should therefore incorporate dose-escalation and schedule-optimization cohorts, pharmacokinetic and pharmacodynamic analyses, serial myeloid biomarker assessments, and adaptive designs that permit treatment modification in response to emerging TAM-resistance states (26, 27, 29, 132, 151).

Delivery and specificity remain substantial barriers. Many TAM-directed targets, including CSF1R, CCR2, PI3Kγ, CD47-SIRPα, AXL, and MerTK, are shared by pathogenic TAMs, circulating monocytes, tissue-resident macrophages, and, in some cases, non-myeloid cells. Consequently, systemic administration of small-molecule inhibitors or antibodies may disrupt host defense, tissue homeostasis, wound repair, or apoptotic-cell clearance. Phagocytosis-checkpoint blockade may also affect healthy target-expressing cells, whereas systemic CD40, TLR, or STING activation may cause excessive inflammatory or cytokine-mediated toxicity. These on-target, off-tumor effects can narrow the clinical safety window. Macrophage-targeted nanoparticles, lipid nanoparticles, antibody-directed carriers, engineered extracellular vesicles, RNA-based therapeutics, bispecific constructs, conditional agents, and local delivery platforms may improve therapeutic selectivity. However, these approaches require careful pharmacodynamic validation to confirm intratumoral accumulation, TAM uptake, target engagement, and functional macrophage reprogramming (28, 313). Companion diagnostics should be developed alongside these agents to verify that the intended macrophage subset is being modified in patients.

Another critical goal is preserving beneficial macrophage functions. Broad macrophage depletion may remove suppressive TAMs, but it can also eliminate macrophages required for antigen presentation, antibody-dependent cellular phagocytosis, tissue repair, host defense, and coordination of antitumor immunity (156). Selective reprogramming may therefore be preferable in tumors where TAMs retain functional plasticity. Depletion strategies should be reserved for tumors dominated by clearly pathogenic macrophage populations or used transiently in combination with immune activation, phagocytosis checkpoint blockade, stromal normalization, or antigen-releasing therapies.

The gut-tumor axis should also be incorporated into future TAM-directed clinical development. Microbiome diversity, antibiotic exposure, diet, microbial metabolites, and enrichment of responder-associated taxa can influence myeloid differentiation, inflammatory tone, antigen presentation, and response to immune checkpoint blockade. Future trials evaluating CSF1R inhibitors, CCR2 antagonists, CD40 agonists, CD47/SIRPα blockers, PI3Kγ inhibitors, or metabolic modulators should consider baseline stool profiling, antibiotic history, and microbiome-derived biomarkers. Microbiome modulation through dietary intervention, prebiotics, probiotics, defined bacterial consortia, microbial metabolites, or fecal microbiota transplantation may provide an additional strategy to enhance macrophage reprogramming and improve immunotherapy responsiveness, although prospective validation remains necessary (41, 222, 314).

The discrepancy between preclinical models and human TAM biology remains a major barrier to clinical translation. Murine transplantable and genetically engineered tumor models often fail to reproduce the ontogenetic diversity, spatial organization, chronic treatment exposure, stromal complexity, metabolic gradients, and interpatient variability of human tumors. Consequently, metabolic inhibitors, polarization modulators, and other TAM-reprogramming agents that produce substantial tumor control in preclinical models may show limited or variable clinical efficacy when the targeted macrophage program is not dominant, is restricted to particular tumor regions, or is replaced by compensatory myeloid states (21, 26, 27, 29, 59, 132, 151). Differences in cancer type, molecular subtype, metastatic site, previous treatment, host metabolism, microbiome composition, and systemic immune status may further modify therapeutic responses. Patient-derived organoids with autologous myeloid co-culture, tumor-on-a-chip systems, 3D-bioprinted stromal models, humanized mouse models, spatially resolved ex vivo tumor slices, and AI-guided multi-omics platforms can more accurately model TAM-tumor-CAF-endothelial interactions, drug penetration, immune exclusion, hypoxia, efferocytosis, angiogenesis, and treatment-induced macrophage remodeling (20, 98, 311, 315). Linking these platforms to patient-specific molecular and spatial biomarkers may improve therapeutic selection and reduce the gap between preclinical activity and clinical benefit.

Future TAM-directed therapy should be guided by several principles. First, the dominant macrophage state and resistance mechanism should be defined before treatment using molecular, spatial, metabolic, and functional biomarkers. Second, therapy should target the specific macrophage program driving resistance rather than applying a uniform TAM-directed approach. Third, TAM modulation should be combined with complementary strategies such as antigen release, immune checkpoint blockade, phagocytosis restoration, vascular normalization, stromal remodeling, metabolic therapy, microbiome modulation, or adoptive cell therapy. Fourth, macrophage adaptation should be monitored during treatment because TAM states can shift rapidly under therapeutic pressure. Finally, clinical endpoints should include not only tumor shrinkage but also the durability of response, prevention of recurrence, metastasis-free survival, immune restoration, and pharmacodynamic evidence of TAM-state conversion (316).

The future of TAM therapeutics largely lies in precision myeloid immuno-oncology. By integrating single-cell and spatial biology, longitudinal monitoring, microbiome assessment, advanced modeling systems, and rational combination therapy, it may become possible to transform TAMs from drivers of immune evasion and therapeutic resistance into active participants in durable antitumor immunity.

### 6.1 Clinical evidence for TAM-targeted and engineered-macrophage therapies

Clinical evidence for TAM- and broader myeloid-directed therapies varies substantially according to disease context, trial design, and therapeutic class (26, 28, 29). The strongest evidence comes from CSF1-driven tenosynovial giant-cell tumor (TGCT), in which randomized phase III trials established the clinical efficacy of CSF1R inhibition with pexidartinib and vimseltinib (317, 318). Emactuzumab also produced high response rates in diffuse-type TGCT in an early-phase study (319). By contrast, CSF1R inhibition showed limited clinical activity in recurrent glioblastoma despite pharmacodynamic evidence of target modulation (320). These findings indicate that activity in a macrophage-dependent disease should not be generalized to heterogeneous solid tumors, in which tissue-resident macrophages, alternative myeloid populations, cancer cells, and stromal components may sustain redundant resistance pathways (26–29, 150).

Early-phase studies of CCR2, PI3Kγ, CD40, and related pathways have demonstrated pharmacodynamic modulation of myeloid cells and, in some settings, preliminary combination activity (321–324). However, the clinical interpretation of these findings remains limited by small cohorts, nonrandomized designs, heterogeneous tumor types, and the use of active partner therapies. For example, mitazalimab plus modified FOLFIRINOX showed promising activity in a single-arm phase II study, whereas the PRINCE trial did not demonstrate a clear benefit for sotigalimab-containing arms (322, 323). CCR2 inhibition reduced circulating CCR2-positive monocytes, and PI3Kγ inhibition demonstrated on-target myeloid reprogramming, but neither finding independently establishes durable antitumor efficacy (321, 324). Randomized, biomarker-guided studies are therefore required before clinical benefit can be attributed specifically to TAM or broader myeloid modulation.

The clinical development of CD47-SIRPα blockade illustrates the distinction between encouraging early-phase activity and confirmed clinical efficacy. Magrolimab combinations produced responses in early single-arm studies of relapsed or refractory non-Hodgkin lymphoma and higher-risk myelodysplastic syndrome (325, 326). However, the randomized phase III ENHANCE trial crossed a prespecified futility boundary and was discontinued, indicating that the earlier activity did not translate into improved clinical outcomes in that setting (327). CD47 expression on erythrocytes and other healthy cells can also create an antigen sink and contribute to anemia and other hematologic toxicities (210–212, 325–327). Moreover, tumor-cell engulfment may not confer durable immunity unless it is coupled with inflammatory antigen presentation and T-cell activation (212, 215). These findings support the development of tumor-selective, conditionally activated, or SIRPα-directed agents and biomarkers that assess functional phagocytosis rather than CD47 expression alone.

Emerging therapies targeting Clever-1/stabilin-1, TREM2, AXL, MerTK, LILRBs, and Siglecs seek to improve selectivity for suppressive or efferocytic macrophage states (26, 42, 150, 212, 235, 328). Early clinical studies of bexmarilimab showed preliminary disease-control and immune-activation signals in selected tumor types, whereas PY314 plus pembrolizumab showed limited antitumor activity in checkpoint-inhibitor-pretreated advanced renal cell carcinoma (26, 328). These findings remain preliminary and should not be interpreted as established efficacy. Receptor expression can vary among lesions and spatial niches, and receptor abundance alone does not demonstrate functional dependence on the targeted pathway (21, 27, 42, 150). Clinical evaluation should therefore assess receptor abundance, macrophage ontogeny, spatial localization, downstream state conversion, and restoration of T-cell or NK-cell activity.

CAR-macrophage and CAR-monocyte therapies offer an alternative approach by engineering myeloid cells to recognize tumor antigens, phagocytose malignant cells, remodel the TME, and support antigen presentation (34, 35, 279). Interim phase I findings for CT-0508 demonstrated manufacturing feasibility, tolerability, intratumoral trafficking, and biological activity, but no objective responses or established survival benefit; the CT-0525 CAR-monocyte study was discontinued before clinical efficacy was established (329, 330). Thus, engineered myeloid cell therapies remain investigational. Antigen heterogeneity, limited cellular persistence, variable tumor penetration, manufacturing complexity, and the risk of suppressive reprogramming within the TME remain major challenges (34, 35, 279, 329). Relevant biomarkers include tumor-antigen density, CAR expression, cellular persistence, intratumoral localization, phagocytic activity, cytokine production, and changes in endogenous T-cell infiltration.

Representative completed, discontinued, and ongoing clinical trials of TAM-targeted, broader myeloid-directed, and engineered-macrophage therapies are summarized in **Table 4**. The table presents key clinical outcomes together with trial-level interpretations that distinguish established randomized efficacy, preliminary early-phase activity, pharmacodynamic modulation, negative or limited findings, and investigational development. Collectively, these studies show that target engagement or macrophage-state modulation does not necessarily translate into objective response, durable disease control, or prolonged survival, particularly in heterogeneous tumors and nonrandomized combination studies (26, 28). Future studies should integrate conventional clinical endpoints-including objective response rate (ORR), duration of response (DoR), progression-free survival (PFS), and overall survival (OS)-with TAM-specific pharmacodynamic measures, including macrophage depletion or state conversion, receptor occupancy, monocyte recruitment, spatial redistribution, antigen presentation, phagocytosis, and restoration of lymphocyte activity. Biomarker-guided enrollment and longitudinal sampling will be necessary to identify patients whose tumors are functionally dependent on the targeted macrophage program (26, 28, 29).

**Table 4:** Representative completed, discontinued, and ongoing clinical trials of TAM-targeted, broader myeloid-directed, and engineered-macrophage therapies.

Trial/NCT number	Phase and status	Cancer type	Agent or intervention	TAM/myeloid-directed mechanism	Molecular target or receptor	Endpoint results	Clinical interpretation	Ref
NCT02371369	Phase III; completed; ENLIVEN	Symptomatic advanced TGCT	Pexidartinib; Turalio	Inhibition of macrophage survival and accumulation	CSF1R	Week-25 ORR: 39% vs. 0% placebo by RECIST	Randomized phase III evidence established the efficacy of CSF1R inhibition in symptomatic, advanced TGCT not amenable to surgery	(317)
NCT05059262	Phase III; completed; MOTION	Symptomatic TGCT	Vimseltinib; Romvimza	Selective inhibition of CSF1R-dependent macrophage accumulation	CSF1R	Week-25 ORR: 40% vs. 0% with placebo; ORR by tumor volume score: 67% in treatment arm	Randomized phase III evidence confirms the efficacy of selective CSF1R inhibition in symptomatic TGCT not amenable to surgery	(318)
NCT04313881	Phase III; discontinued; ENHANCE	Previously untreated higher-risk MDS	Magrolimab plus azacitidine versus azacitidine	CD47-SIRPα checkpoint blockade	CD47-SIRPα; cytogenetic and molecular risk markers	Discontinued after crossing a prespecified futility boundary; the study was unlikely to demonstrate benefit in CR and OS	Negative phase III results showed that promising early-phase activity did not translate into improved clinical outcomes	(327)
NCT01349036	Phase II; completed	Recurrent glioblastoma	Pexidartinib or PLX3397	Inhibition of CSF1R-positive glioma-associated macrophages and microglia	CSF1R	No objective responses; 6-month PFS: 8.8%; median OS: 9.4 months	Despite pharmacodynamic effects, CSF1R inhibition alone showed limited clinical activity in recurrent glioblastoma	(320)
NCT04888312	Phase II; completed; OPTIMIZE-1	First-line metastatic PDAC	Mitazalimab plus modified FOLFIRINOX (ADC-1013)	CD40-mediated activation of macrophages and other antigen-presenting cells	CD40	Confirmed ORR: 42.1%; median DoR: 12.6 months; median PFS: 7.7 months; median OS: 14.9 months	Single-arm phase II results showed promising activity, but efficacy requires confirmation in a randomized trial	(323)
NCT01413022	Phase Ib; completed	Borderline-resectable or locally advanced PDAC	PF-04136309 plus FOLFIRINOX	Reduction in inflammatory-monocyte recruitment	CCR2	Partial response rate: ≈49% (16/33); local tumor control; DCR: 97% (32/33); reduced circulating CCR2-positive monocytes	Early-phase results support the feasibility and potential activity of CCR2 inhibition with FOLFIRINOX; randomized confirmation is required	(321)
NCT02637531	Phase I/Ib; completed; MARIO-1	Advanced solid tumors, including immune-checkpoint inhibitor-resistant cancers, HNSCC, TNBC, NSCLC, melanoma, adrenocortical carcinoma, mesothelioma	Eganelisib or IPI-549 alone or with nivolumab (Opdivo)	Functional reprogramming of suppressive myeloid cells	PI3K-gamma	Manageable safety and early activity, including in resistant disease	Demonstrated on-target myeloid reprogramming and preliminary combination activity; efficacy was not established in this nonrandomized phase I/Ib study	(324)
NCT03214250	Phase Ib/2; completed; PRINCE	Metastatic PDAC	Sotigalimab or APX005M plus gemcitabine nab-paclitaxel, with or without nivolumab	Activation of macrophages, dendritic cells, and other antigen-presenting cells	CD40	1-year OS: 57.7% with nivolumab plus chemotherapy, 48.1% with sotigalimab plus chemotherapy, and 41.3% with the triple-combination	The primary 1-year OS endpoint was met only by nivolumab plus chemotherapy; no clear benefit was demonstrated for sotigalimab-containing arms	(322)
NCT02953509	Phase Ib/2; completed	Relapsed or refractory NHL	Magrolimab (Hu5F9-G4) plus rituximab	Restoration of macrophage-mediated phagocytosis with antibody-mediated opsonization	CD47-SIRPα and CD20	ORR: 52.2%; CR rate: 30.4%; DOR: 15.9 months; PFS: 7.4 months; OS: not reached	Durable activity was observed in relapsed/refractory indolent NHL, but randomized confirmation is needed	(325)
NCT03248479	Phase Ib; (5F9005), completed	Higher-risk myelodysplastic syndrome, hematologic malignancies (blood cancers), AML, MDS	Magrolimab (Hu5F9-G4) plus azacitidine	Restoration of macrophage phagocytosis	CD47-SIRPα	ORR: 75%; CR: 33%; median time to response: 1.9 months; median duration of CR: 11.1 months; median OS not reached at 17.1 months’ follow-up	Promising single-arm phase Ib activity was not confirmed in the subsequent randomized phase III ENHANCE trial	(326)
NCT01494688	Phase I/Ib; completed	dTGCT/PVNS, advanced and/or metastatic solid tumors	Emactuzumab; RO5509554 or RG7155	Depletion of CSF1R-positive macrophages	CSF1R	ORR by RECIST: 71%; DCR: 98% in patients with diffuse-type TGCT	Emactuzumab produced high and durable response rates in CSF1-dependent diffuse-type TGCT; these results should not be generalized to the study’s other solid-tumor cohorts	(319)
NCT03733990	Phase I/II; MATINS; completed	Advanced refractory solid tumors	Bexmarilimab	Conversion of suppressive macrophages toward inflammatory antigen-presenting states	Clever-1 or stabilin-1	DCR: 25%-40% across selected cancer cohorts	Bexmarilimab showed preliminary disease-control and immune-activation signals in selected tumor types; biomarker-guided randomized validation is required	(26)
NCT04691375	Phase I/II; completed	Locally advanced (unresectable) and/or metastatic solid tumors, ccRCC, breast, ovarian, colorectal, and lung adenocarcinoma	PY314 alone or with pembrolizumab	Targeting of TREM2-positive suppressive macrophages	TREM2	In checkpoint-inhibitor-pretreated advanced RCC: ORR 5.9% (1/17); DCR 29.4% (5/17); median PFS 1.4 months; grade ≥3 TRAEs 5.9%	PY314 plus pembrolizumab was tolerable but showed limited antitumor activity in checkpoint-inhibitor-pretreated advanced RCC	(328)
NCT04660929	Phase I; active, not recruiting	HER2-overexpressing recurrent or metastatic solid tumors	CT-0508 autologous anti-HER2 CAR-M; or CT-0508 with Pembrolizumab (Keytruda)	HER2-directed recognition, phagocytosis, antigen presentation, and TME remodeling	HER2-directed CAR-M	No DLTs, grade ≥3 CRS, or ICANS; no objective responses. At 8 weeks, SD occurred in 4/9 (44%) patients with HER2 3+ tumors; all 5/5 patients with HER2 2+ tumors had PD	Interim phase I findings demonstrated manufacturing feasibility, tolerability, tumor trafficking, and biological activity; objective response and survival benefit were not established	(329)
NCT06254807	Phase I; discontinued	HER2-overexpressing advanced or metastatic solid tumors	CT-0525 anti-HER2 CAR-monocyte	Engineered monocytes traffic to tumors and differentiate into CAR-M	HER2-directed CAR-monocyte	No clinical efficacy results publicly reported	Clinical efficacy was not established before study discontinuation	(330)

RECIST, response evaluation criteria in solid tumors, ORR, overall response rate, OS, overall survival, PFS, progression-free survival, CR, complete response, DoR, duration of response, DCR, disease control rate, TRAE, treatment-related adverse events, HNSCC, head and neck squamous cell carcinoma, TNBC, triple-negative breast cancer, NSCLC, non-small cell lung cancer, MDS, myelodysplastic syndrome, TGCT, tenosynovial giant-cell tumor, PDAC, pancreatic ductal adenocarcinoma, NHL, non-Hodgkin lymphoma, AML, acute myeloid leukemia, dTGCT/PVNS, diffuse-type tenosynovial giant-cell tumor, ccRCC, clear cell renal cell carcinoma, CAR-M, chimeric antigen receptor-macrophage, DLT, dose-limiting toxicity; CRS, cytokine release syndrome; ICANS, immune effector cell-associated neurotoxicity syndrome; SD, stable disease; PD, progressive disease.

## 7 Conclusion

TAMs are central regulators of tumor initiation, malignant progression, immune evasion, metastasis, and therapeutic resistance. Their functions are determined by developmental origin, tumor type, disease stage, anatomical niche, recruitment pathways, cytokine and growth-factor signaling, hypoxia, metabolism, epigenetic regulation, ECM architecture, stromal interactions, extracellular vesicles, microbiome-derived signals, and treatment exposure. TAMs, therefore, cannot be adequately classified as a uniform M2-like population. Instead, they comprise spatially organized and interconverting inflammatory, antigen-presenting, angiogenic, lipid-associated, efferocytic, matrix-remodeling, and immunosuppressive states (21). These context-dependent states have distinct biological functions and therapeutic vulnerabilities (28).

Bidirectional cancer-TAM crosstalk evolves throughout tumor development and treatment. Cancer cells recruit and educate macrophages through cytokines, chemokines, metabolites, and extracellular vesicles, whereas TAMs promote tumor-cell survival, stemness, invasion, immune evasion, and drug resistance. Therapy-resistant cancer cells can further expand suppressive TAM populations, which protect residual disease through lymphocyte suppression, tolerogenic efferocytosis, angiogenesis, metabolic restriction, stromal remodeling, and regenerative niche formation. These interactions vary among malignancies: relatively inflammatory or antigen-presenting macrophage states may persist in some lung cancers (80), whereas PDAC is commonly dominated by hypoxic, fibrotic, and strongly immunosuppressive TAM programs (155). TAM-directed therapy must therefore be matched to tumor type, disease stage, macrophage ontogeny, spatial niche, and dominant resistance mechanism.

Therapeutic development has progressed from broad macrophage depletion toward selective reprogramming, recruitment blockade, innate activation, restoration of phagocytosis, modulation of efferocytosis, metabolic and epigenetic intervention, stromal or vascular normalization, targeted delivery, and engineered macrophage or monocyte therapies. Clinical trials demonstrate that TAM targeting can produce meaningful activity when macrophage dependence is a defining feature of disease, as observed with CSF1R-directed therapy in tenosynovial giant-cell tumor (317). However, limited responses in heterogeneous solid tumors, including recurrent glioblastoma, and inconsistent findings from several early macrophage-directed approaches show that target engagement or TAM-state modification does not necessarily translate into durable tumor regression or prolonged survival (320).

Clinical progress will require overcoming spatial and cellular heterogeneity, adaptive TAM plasticity, compensatory myeloid pathways, off-target toxicity, and discrepancies between preclinical models and human tumors (26). Combination regimens must also be optimized for dose, sequence, timing, duration, and overlapping toxicity. Single-cell and spatial profiling, longitudinal monitoring, functional macrophage biomarkers, human-relevant experimental models, and companion diagnostics should guide patient selection and confirm durable TAM-state conversion. Ultimately, precision myeloid immuno-oncology, rather than uniform macrophage targeting, offers the strongest opportunity to redirect pathogenic TAM programs while preserving beneficial macrophage functions and improving the durability of anticancer therapy (29).
